# Nidogen 1‐Enriched Extracellular Vesicles Facilitate Extrahepatic Metastasis of Liver Cancer by Activating Pulmonary Fibroblasts to Secrete Tumor Necrosis Factor Receptor 1

**DOI:** 10.1002/advs.202002157

**Published:** 2020-08-19

**Authors:** Xiaowen Mao, Sze Keong Tey, Cherlie Lot Sum Yeung, Ernest Man Lok Kwong, Yi Man Eva Fung, Clive Yik Sham Chung, Lung‐Yi Mak, Danny Ka Ho Wong, Man‐Fung Yuen, James Chung Man Ho, Herbert Pang, Maria Pik Wong, Carmen Oi‐Ning Leung, Terence Kin Wah Lee, Victor Ma, William Chi‐Shing Cho, Peihua Cao, Xiaoping Xu, Yi Gao, Judy Wai Ping Yam

**Affiliations:** ^1^ Department of Pathology, Li Ka Shing Faculty of Medicine The University of Hong Kong Hong Kong; ^2^ Department of Chemistry, State Key Laboratory of Synthetic Chemistry The University of Hong Kong Pokfulam Hong Kong; ^3^ School of Biomedical Sciences, Li Ka Shing Faculty of Medicine The University of Hong Kong Hong Kong; ^4^ Department of Medicine, Li Ka Shing Faculty of Medicine The University of Hong Kong Hong Kong; ^5^ State Key Laboratory of Liver Research The University of Hong Kong Hong Kong; ^6^ School of Public Health, Li Ka Shing Faculty of Medicine The University of Hong Kong Hong Kong; ^7^ Department of Applied Biology and Chemical Technology The Hong Kong Polytechnic University Kowloon Hong Kong; ^8^ Department of Clinical Oncology Queen Elizabeth Hospital Kowloon Hong Kong; ^9^ Department of Hepatobiliary Surgery II, Zhujiang Hospital Southern Medical University Guangzhou 510280 China; ^10^ Clinical Research Center Zhujiang Hospital Southern Medical University Guangzhou 510280 China; ^11^ Guangdong Provincial Research Center of Artificial Organ and Tissue Engineering, Zhujiang Hospital Southern Medical University Guangzhou 510280 China

**Keywords:** extracellular vesicles, hepatocellular carcinoma, nidogen 1, pre‐metastatic niche, tumor necrosis factor receptor 1

## Abstract

In hepatocellular carcinoma (HCC) patients with extrahepatic metastasis, the lung is the most frequent site of metastasis. However, how the lung microenvironment favors disseminated cells remains unclear. Here, it is found that nidogen 1 (NID1) in metastatic HCC cell‐derived extracellular vesicles (EVs) promotes pre‐metastatic niche formation in the lung by enhancing angiogenesis and pulmonary endothelial permeability to facilitate colonization of tumor cells and extrahepatic metastasis. EV‐NID1 also activates fibroblasts, which secrete tumor necrosis factor receptor 1 (TNFR1), facilitate lung colonization of tumor cells, and augment HCC cell growth and motility. Administration of anti‐TNFR1 antibody effectively diminishes lung metastasis induced by the metastatic HCC cell‐derived EVs in mice. In the clinical perspective, analysis of serum EV‐NID1 and TNFR1 in HCC patients reveals their positive correlation and association with tumor stages suggesting the potential of these molecules as noninvasive biomarkers for the early detection of HCC. In conclusion, these results demonstrate the interplay of HCC EVs and activated fibroblasts in pre‐metastatic niche formation and how blockage of their functions inhibits distant metastasis to the lungs. This study offers promise for the new direction of HCC treatment by targeting oncogenic EV components and their mediated pathways.

## Graphical abstract

Nidogen 1‐enriched extracellular vesicle (EV‐NID1) activates pulmonary fibroblasts to secrete tumor necrosis factor receptor 1 (TNFR1) which favors lung metastasis of liver cancer. Serum EV‐NID1 and TNFR1 are potential noninvasive biomarkers for the early detection of liver cancer. This study also provides insights into a new direction of treatment by blocking the signals of EVs derived from tumor cells.

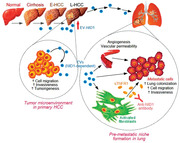

## 1 Introduction

Intercommunication between tumor cells and their microenvironment plays a crucial role during cancer development and metastasis.^[^ ^1^ ^]^ Extracellular vesicle (EV) shedding has emerged as an important channel for intercellular communication. The distinct functional properties of EVs are determined by the composition of lipids, proteins, and RNAs present within the EVs, thus resulting in EVs with numerous potential functions.^[^ ^2^ ^]^

Long‐range signaling between local tumor cells and distant cells can be mediated by the transport of functional oncogenes through tumor‐derived exosomes, which subsequently influences the signaling and behavior of recipient cells.^[^ ^3^, ^4^ ^]^ Evidence in different cancer models has revealed the role of tumor‐derived exosomes in generating a pre‐metastatic niche that favors the survival of disseminated tumor cells in distant organs. EVs released by chronic lymphocytic leukemia cells induce the phenotype of cancer‐associated fibroblasts in stromal cells, which contributes to a tumor‐supportive microenvironment.^[^ ^5^ ^]^ Another study showed that EVs derived from pancreatic ductal adenocarcinoma initiate pre‐metastatic niche formation and consequently increase liver metastatic burden in naïve mice.^[^ ^6^ ^]^ EVs from highly metastatic melanomas are able to educate bone marrow progenitors through the upregulation of Met and induce vascular leakiness at pre‐metastatic sites.^[^ ^7^ ^]^ These studies highlight the multifaceted roles of tumor‐derived EVs in the modulation of the tissue microenvironment to facilitate metastasis.

The tumor microenvironment is influenced by numerous stromal factors that coordinate to provide a nourishing condition for cancer cells to survive and grow. Cancer‐associated fibroblasts (CAFs) are one of the most prominent stromal components in the tumor microenvironment. Compelling evidence has documented the activity of CAFs in enhancing tumor formation and metastasis in different ways. CAFs have been shown to recruit proinflammatory immune cells and enhance angiogenesis in tumors.^[^ ^8^ ^]^ CAFs are pivotal players that contribute to an immunosuppressive tumor microenvironment.^[^ ^9^, ^10^ ^]^ Cancer stem cells are capable of mediating cancer metastasis and drug resistance. Conditioned medium from CAF cultures has been shown to activate cancer stemness properties of cancer cells via paracrine secretion of hepatocyte growth factor.^[^ ^11^ ^]^ It has been shown that blocking the communication between cancer cells and CAFs significantly diminishes hepatocellular carcinoma (HCC) growth and dissemination.^[^ ^12^ ^]^ As a robust marker of activated fibroblasts, alpha‐smooth muscle actin (*α*‐SMA) in the stroma of multiple solid tumors identifies patients with poor survival.^[^ ^13^ ^]^ In HCC, *α*‐SMA is present in the majority of metastatic lesions.^[^ ^14^ ^]^ HCC cases with high *α*‐SMA staining in the peritumoral region have a significantly worse prognosis than HCC cases with low peritumoral *α*‐SMA signal.^[^ ^15^ ^]^

HCC accounts for most liver cancers and is currently the third leading cause of cancer‐related death worldwide. Metastasis is a key event at the advanced stage of hepatocarcinogenesis. Approximately half of the patients with extrahepatic HCC are presented with metastasis in the lung, the most frequent site of extrahepatic HCC.^[^ ^16^ ^]^ However, it remains unclear how the microenvironment in the lungs favors incoming metastatic cells. Therefore, we endeavored to determine how HCC‐derived EVs activate pulmonary fibroblasts to cultivate a supportive microenvironment for disseminated metastatic cells to colonize the lungs.

## 2 Results

### 2.1 EV Secretion from Metastatic HCC Cells Promotes Liver Tumor Formation and Metastasis to the Lungs

**Figure 1:**
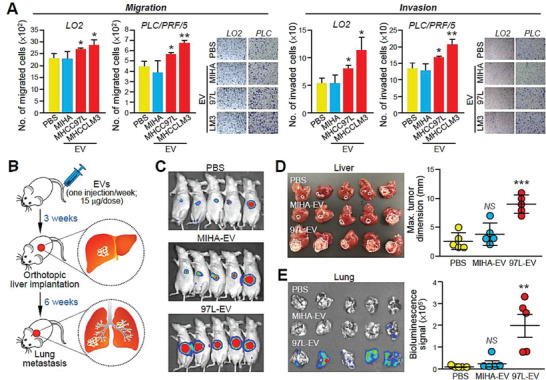
EVs from metastatic MHCC97L cells promote HCC tumorigenesis and metastasis. A) Migration and invasion assays of LO2 and PLC/PRF/5 cells pretreated with EVs derived from MIHA, MHCC97L, or MHCCLM3 cells. Cells treated with PBS were included as controls. Representative images of fixed and crystal violet‐stained migrated and invaded cells at the end of the experiment are shown. B) Schematic diagram of the EV education mouse model. Nude mice were injected with EVs derived from MIHA or MHCC97L cells via tail vein once a week for 3 weeks (15 µg per week) prior to orthotopic liver implantation of tumor seeds derived from naïve luciferase‐labeled MHCC97L cells (*n* = 5). Analysis of liver tumors formed and distant lung metastases was performed 5 weeks after liver implantation. C) Bioluminescence imaging of animals at the end of the experiment. D) Image of excised livers. Measurement of liver tumor size is plotted. E) Bioluminescence imaging of dissected lung tissues. Quantification of the luciferase signal is shown. Three independent experiments were performed in triplicate for assays shown in (A) and (B). Data are represented as the mean ± SEM; :*p* < 0.05; ::*p* < 0.01; :::*p* < 0.001; NS, not significant from Student's *t*‐test.

The properties of EVs derived from the metastatic HCC cell lines MHCC97L and MHCCLM3 (MHCC97L‐ and MHCCLM3‐EVs) and their effects on target cells were investigated. Both cell lines were established from metastatic lesions of HCC patients with lung metastasis.^[^ ^17^ ^]^ EVs from the immortalized normal liver cell line MIHA (MIHA‐EVs) were included for functional comparison. The size, integrity, and purity of the isolated EVs were validated (Figure S1A,B,D, Supporting Information). The relative amount of EV protein obtained was significantly higher in the medium of metastatic cells than in the medium of normal cells (Figure S1C, Supporting Information). EVs from metastatic cells significantly augmented both the migratory and invasive properties of naïve LO2 liver cells and PLC/PRF/5 HCC cells (Figure**1A**). Internalization of PKH26‐labeled EVs was observed in recipient cells after incubation (Figure S2, Supporting Information).

Using an EV education mouse model^[^ ^6^ ^]^ comprising the repeated EV injections prior to the implantation of HCC tumor seeds in the liver (Figure 1B), we observed that compared to mice treated with MIHA‐EVs, mice administered with MHCC97L‐EVs showed enhanced growth of the primary tumor in the liver (Figure 1C,D) and increased distant metastasis to the lungs (Figure 1E). Tipifarnib, a farnesyltransferase inhibitor, was identified as a potent inhibitor of EV biogenesis and secretion.^[^ ^18^ ^]^ Tipifarnib has been shown to inhibit tumorigenesis of thyroid and breast cancers.^[^ ^19^, ^20^ ^]^ In our mouse model, treatment with tipifarnib reduced EV secretion from MHCC97L cells by up to 50% (Figure S3A, Supporting Information). Injection of MHCC97L cells in the livers of mice pretreated with tipifarnib resulted in a significant delay in tumor development (Figure S3B, Supporting Information). These data suggest that EV secretion is crucial to liver tumor formation and distant metastasis.

### 2.2 Metastatic HCC‐Derived EVs Promote Angiogenesis and Facilitate the Colonization of Tumor Cells in the Lungs

**Figure 2:**
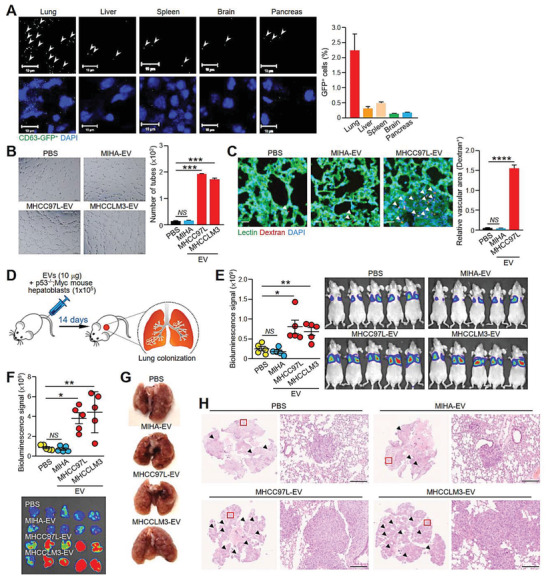
EVs from metastatic MHCC97L cells enhance endothelial leakiness and hepatoblasts colonization in the lungs. A) Tissue distribution of EVs in tissues of mice. 24 h after mice were intravenously injected with EVs derived from MHCC97L CD63‐GFP cells, the mice were subjected to euthanasia, perfused, and fixed for 24 h before tissue dissection. Tissue sections were examined under confocal microscopy. Black and white images reveal the detection of EVs in different tissues (upper panel). EVs are indicated by the arrowhead. Images of GFP^+^ EVs and DAPI‐stained nuclei are shown (lower panel). Quantification of the signal in five random fields of three tissue sections per organ is shown. Scale bar: 10 µm. B) Tube formation assay of HUVECs pretreated with the indicated EVs. Quantification of capillary‐like tubular structures formed is shown. C) Analysis of lung vessel leakiness after tail vein injection of MHCC97L‐EVs, Texas Red‐Dextran, and FITC‐Lectin. The arrowhead indicates the area of endothelial leakiness. Scale bar: 20 µm. D) Analysis of lung colonization of murine p53−/−; Myc hepatoblasts (1 × 10^5^) 2 weeks after coinjection with the indicated EVs (10 µg) via tail vein (*n* = 5). E) Image of bioluminescence signals of mice at the end of the experiment. Quantification of the luciferase signal is shown. F) Bioluminescence imaging of dissected lung tissues and quantification of the luciferase signal. G) Representative image of a dissected lung after fixation. H) Representative images of hematoxylin and eosin (H&E) staining of lung tissues. Examples of metastatic lesions are indicated by arrowheads. Insets show the enlarged area of the metastatic lesions. Magnification, 5 ×; Scale bar, 200 µm. Three independent experiments were performed in triplicate for assays shown in (A)–(C). Data are represented as the means ± SEM; :*p* < 0.05; ::*p* < 0.01; :::*p* < 0.001; ::::*p* < 0.0001; NS, not significant from Student's *t*‐test.

We further explored how EVs cultivate a supportive microenvironment to facilitate metastasis. Intravenously injected MHCC97L‐derived EVs labeled with CD63‐GFP or PKH67 were predominantly localized in the lungs and livers of mice (Figure 2A and Figure S4, Supporting Information). Destabilization and increased permeability of the vasculature in the lungs are early events in pre‐metastatic niche formation.^[^ ^21^ ^]^ Indeed, compared to MIHA‐EVs, MHCC97L‐ and MHCCLM3‐EVs promoted human umbilical vein endothelial cells (HUVECs) to form capillary‐like structures (Figure 2B). MHCC97L‐EVs also enhanced the pulmonary endothelial permeability in mice, as indicated by the larger area of dextran staining (Figure 2C). Coinjection of murine p53−/−; Myc‐transduced hepatoblasts with MHCC97L‐ and MHCCLM3‐EVs but not MIHA‐EVs in mice resulted in enhanced colonization of hepatoblasts to lungs as revealed by the elevated bioluminescence signals and formation of tumor nodules in the lungs (Figure 2D–G). Histological examination of the lungs revealed a profound increase in the number of metastatic lesions in mice injected with MHCC97L‐ and MHCCLM3‐EVs (Figure 2H).

### 2.3 The EV‐NID1 Level is Positively Correlated with the Metastatic Potential of Parental Cells

**Figure 3:**
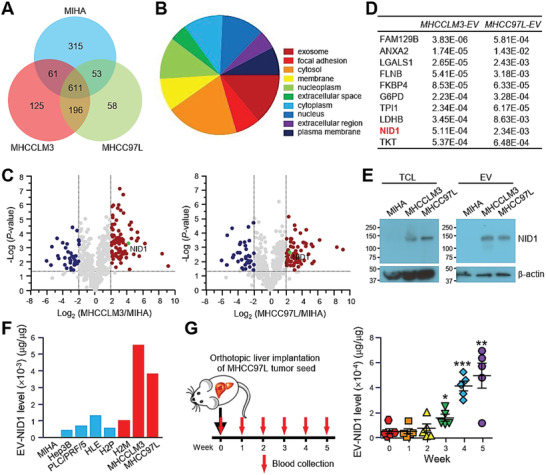
NID1 level in EVs correlates with the metastatic potential of cells and tumor burden in mice. A) Protein was extracted from EVs derived from MIHA, MHCCLM3, and MHCC97L cells and was subjected to mass spectrometry analysis (technical triplicate/sample). Venn diagram illustrating the number of proteins that were commonly and uniquely expressed in EVs of the indicated cell lines. B) Analysis of the distribution of cellular components of proteins commonly identified in MHCCLM3‐ and MHCC97L‐EVs using FunRich3.1.3. C) Volcano plots of proteins that were significantly modulated by at least fourfold in MHCCLM3‐EVs (left) and MHCC97L‐EVs (right) when compared to proteins of MIHA‐EVs with *p*‐value < 0.05. D) Top ten upregulated proteins identified in MHCCLM3‐EVs ranked by *p*‐value. Significance of their upregulation in MHCC97L‐EVs is listed accordingly. E) Immunoblots showing NID1 expression in the total cell lysate (TCL) and EVs of MIHA, MHCCLM3, and MHCC97L cells. F) Analysis of NID1 expression in EVs derived from MIHA cells and different HCC cell lines was performed in duplicate by ELISA. G) Collection of blood from mice before and after orthotopic liver implantation of luciferase‐labeled MHCC97L tumor seed (*n* = 5). EVs were isolated from the serum and subjected to protein extraction. Serum EV‐NID1 level was analyzed in duplicate using ELISA. Data are represented as the mean ± SEM; :*p* < 0.05; ::*p* < 0.01; :::*p* < 0.001 from Student's *t*‐test.

To comprehensively elucidate the differential biological activity of EVs, proteomic compositions of MIHA‐, MHCC97L‐, and MHCCLM3‐EVs were determined using mass spectrometry (Table S1, Supporting Information). A total of 1040, 995, and 918 proteins were identified in EVs of MIHA, MHCCLM3, and MHCC97L cells, respectively (Figure **3**A). Among these proteins, 611 were commonly identified between EVs of three cell lines irrespective of their metastatic potentials. MHCCLM3‐ and MHCC97L‐EVs shared 807 common proteins which were at least three times more than those detected within MIHA‐EVs. Major cellular distribution of these common proteins in exosome, focal adhesion, and cytosol was analysized by FunRich software (Figure 3B). Volcano plots revealed EVs proteins of MHCCLM3 and MHCC97L cells that were differentially expressed by at least fourfold when compared to EVs of normal liver cells with *p*‐value less than 0.05 (Figure 3C). In total, 118 and 115 significantly modulated proteins were identified in MHCCLM3‐ and MHCC97L‐EVs, respectively. Top ten significantly upregulated EV proteins of MHCCLM3 were shown in Figure 3D. Among which, NID1 ,which has accumulating evidence about its role in cancer, was chosen for further investigation. NID1 is a major structural protein of the basement membrane and extracellular matrix (ECM). Its role in cancer metastasis has been described in ovarian and endometrial cancer;^[^ ^22^, ^23^, ^24^ ^]^ however, its role in HCC remains uncertain, and the significance and existence of NID1 in EVs of HCC have never been reported. NID1 expression in total cell lysate and in EVs from MHCC97L and MHCCLM3 cells but not from MIHA cells was validated (Figure 3E). The level of EV‐NID1 also correlated well with the metastatic potential of HCC cells (Figure 3F). In a mouse model of liver implantation of MHCC97L cells in which the development of liver tumors was monitored weekly by bioluminescence imaging (Figure S5A–C, Supporting Information), the level of NID1 in circulating EVs was higher in tumor‐bearing mice than in mice prior to tumor cell inoculation and increased with the intensity of bioluminescence (Figure 3G and Figure S5, Supporting Information). These findings indicate that the level of EV‐NID1 reflects the metastatic ability of cells and tumor burden in mice.

### 2.4 EV‐NID1 Promotes Pre‐Metastatic Niche Formation and Distant Metastasis to the Lungs

**Figure 4:**
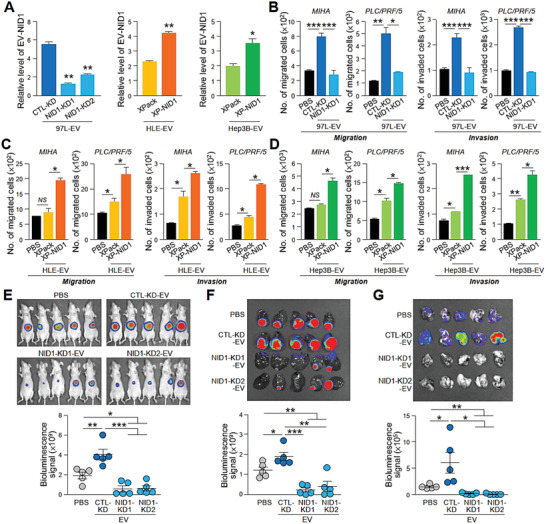
EV‐NID1 is a functional component that drives HCC motility, tumorigenesis, and metastasis. A) ELISA analysis of NID1 levels in EVs derived from MHCC97L (97L) control (CTL‐KD) and NID1 knockdown cells (NID1‐KD1 and NID1‐KD2) and control (XPack) and NID1 overexpressing cells (XP‐NID1) established in HLE and Hep3B cells. The analysis was performed in triplicate. B) Examination of the migratory potential and invasiveness of MIHA and PLC/PRF/5 cells pretreated with MHCC97L CTL‐KD‐ and NID1‐KD‐EVs. C) Examination of the migratory potential and invasiveness of MIHA and PLC/PRF/5 cells pretreated with HLE XPack‐ and XP‐NID1‐EVs. D) Examination of the migratory potential and invasiveness of MIHA and PLC/PRF/5 cells pretreated with Hep3B XPack‐ and XP‐NID1‐EVs. E) EV mouse model comparing the effects of EVs from MHCC97L CTL‐KD and NID1‐KD cells on HCC tumorigenesis and metastasis (*n* = 5). Image showing the luciferase signal of the animals at the end of the experiment. Quantification of the luciferase signal is shown. F) Bioluminescence imaging of dissected liver tissues. Quantification of the luciferase signal is shown. G) Bioluminescence imaging of dissected lung tissues. Quantification of the luciferase signal is shown. Three independent experiments were performed in triplicate for assays shown in (C) and (D). Data are represented as the mean ± SEM; :*p* < 0.05; ::*p* < 0.01; :::*p* < 0.001; NS, not significant from Student's *t*‐test.

To determine the role of NID1 in HCC, NID1 expression was knocked down in MHCC97L cells and engineered to be expressed in EVs from Hep3B and HLE cells using an expression vector with an EV targeting signal (Figure S6A,D, Supporting Information). Knockdown of NID1 resulted in diminished ability of the tumor cells to grow, migrate, and invade (Figure S6B,C, Supporting Information). Conversely, overexpression of EV‐NID1 showed opposing effects (Figure S6E–H, Supporting Information). EVs collected from these stable clones were validated (Figure S7, Supporting Information). The reduced NID1 level in EVs from cells with NID1 knockdown (NID1‐KD1 and NID1‐KD2) and elevated NID1 level in EVs from cells overexpressing NID1 (XP‐NID1) were revealed by enzyme‐linked immunosorbent assay (ELISA) (Figure 4A) and immunoblotting (Figure S7B, Supporting Information). Treatment with EVs from nontarget control cells (CTL‐KD‐EVs) enhanced the migration and invasion ability of naïve cells. However, this enhancement was abolished in cells treated with NID1‐KD‐EV (Figure 4B). XP‐NID1‐EVs displayed a more potent effect on promoting cell motility and invasiveness than did EVs derived from vector control cells (XPack‐EVs) (Figure 4C,D). Consistent with the effect of parental MHCC97L‐EVs, CTL‐KD‐EVs demonstrated the positive effect on liver tumor formation and distant metastasis in the EV treatment model. The augmented metastasis to the lungs was not observed in mice injected with NID1‐KD‐EVs (Figure 4E–G). These functional characterizations provide evidence about the imperative activity of EV‐NID1 in tumor growth and metastasis.

**Figure 5:**
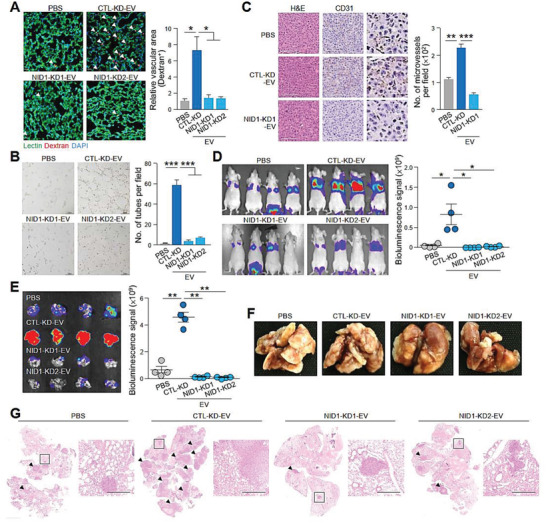
EVs with reduced NID1 levels show a diminished ability to increase vascular permeability, enhance angiogenesis, and facilitate colonization of hepatoblasts in the lung. A) Analysis of lung vessel leakiness after tail vein injection of PBS, MHCC97L CTL‐KD‐EVs, or NID1‐KD‐EVs; Texas Red‐Dextran and FITC‐Lectin. The arrowhead indicates the area of endothelial leakiness. Scale bar: 20 µm. B) Tube formation of HUVECs pretreated with EVs. Quantification of the capillary‐like tubular structures formed is shown. Three independent experiments were performed in triplicate. C) In vivo angiogenesis plug formation assay performed by subcutaneous coinjection of PLC/PRF/5 cells with PBS, MHCC97L CTL‐KD‐EVs, or NID1‐KD‐EVs. Representative images showing H&E staining and immunohistochemistry of dissected tumors using anti‐CD31 antibody are shown. The inset shows the enlarged area of the tumors. Scale bar: 100 µm. The number of microvessels is counted. D) Analysis of lung colonization of murine p53−/−; Myc hepatoblasts (1 × 10^5^) after coinjection with EVs (10 µg) via tail vein (*n* = 4). Bioluminescence imaging of mice at the end of the experiment. Quantification of the luciferase signal is shown. E) Bioluminescence imaging of dissected lung tissues. Quantification of the luciferase signal is shown. F) Representative image of dissected lung after fixation. G) Representative images of H&E staining of lung tissues. Examples of metastatic lesions are indicated by arrowheads. Insets show the enlarged area of the metastatic lesions. Magnification, 2.5 ×; Scale bar, 500 µm. Data are represented as the mean ± SEM; :*p* < 0.05; ::*p* < 0.01; :::*p* < 0.001; NS, not significant from Student's *t*‐test.

The role of EV‐NID1 in modulating the microenvironment in lungs was examined. MHCC97L CTL‐KD‐EVs enhanced vascular permeability when compared to untreated mice. However, the enhancing effect was not observed in mice injected with NID1‐KD‐EVs (Figure 5A). In addition, NID1 knockdown cells resulted in the release of EVs that abolished the promotion of tube‐like structure formation of HUVECs and microvessel formation in the matrigel plug angiogenesis assay (Figure 5B,C), while XP‐NID1‐EVs promoted the formation of tube‐like structures in endothelial cells (Figure S8A, Supporting Information). Compared to cells injected with phosphate‐buffered saline (PBS), mice injected with p53−/−; Myc hepatoblasts and CTL‐KD‐EVs showed a profound increase in the colonization of hepatoblasts to the lungs, whereas the colonization of hepatoblasts in the lungs was largely diminished in mice injected with NID1‐KD‐EVs (Figure 5D–G). Conversely, in mice injected with XP‐NID1‐EVs, augmented colonization of hepatoblasts in the lungs was observed (Figures S8B and S9D, Supporting Information). Taken together, these findings suggest the role of EV‐NID1 in destabilizing the vascular architecture and promoting angiogenesis in the lung, thereby facilitating tumor cell colonization.

### 2.5 EV‐NID1 Activates Pulmonary Fibroblasts to Secrete TNFR1

**Figure 6:**
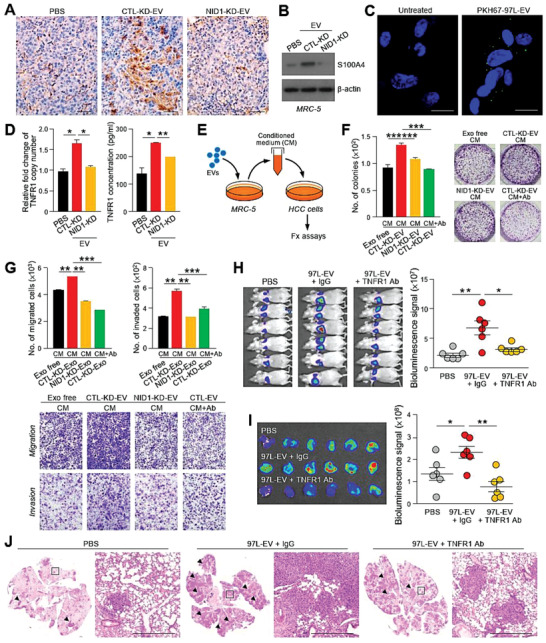
TNFR1 secreted by EV‐NID1‐activated pulmonary fibroblasts promotes HCC cell motility and colonization in the lungs. A) Immunohistochemistry of metastatic lesions in lungs tissues obtained from mice injected with PBS, MHCC97L CTL‐KD‐EVs of NID1‐KD‐EVs using anti‐*α*‐SMA antibody. Magnification, 20 ×; Scale bar, 25 µm. B) Immunoblotting of S100A4 expression in MRC‐5 cells treated with EVs for 24 h. C) Immunofluorescence in MRC‐5 cells after a 24 h incubation with PKH67‐labeled MHCC97L‐EVs. Scale bar: 20 µm. D) Analysis of TNFR1 copy number and concentration of soluble TNFR1 in MRC‐5 cells pretreated with the indicated EVs using qPCR and ELISA, respectively. E) Diagram illustrating the collection of conditioned medium from MRC‐5 cells pretreated with EVs for functional assays. F) Colony formation assay performed with Hep3B incubated with the conditioned medium from MRC‐5 cells incubation with EVs from CTL‐KD or NID1‐KD cells for 72 h. Anti‐TNFR1 neutralizing antibody was added to neutralize the activity of soluble TNFR1 (Ab) (0.4 µg mL^−1^) in the conditioned medium. Representative image shows the fixed and crystal violet‐stained colonies. G) Migration and invasion assays performed using PLC/PRF/5 cells pretreated as described in (F). Representative image shows the fixed and crystal violet‐stained migratory and invasive cells. H) Bioluminescence imaging of mice (*n* = 6) subjected to intravenous coinjection of murine p53−/−; Myc hepatoblasts (1 × 10^5^) with PBS, IgG (10 µg), or anti‐TNFR1 antibody (TNFR1 Ab) (10 µg). Quantification of the luciferase signal is shown. I) Ex vivo bioluminescence imaging of lung tissues. Quantification of the luciferase signal is shown. J) Representative images of H&E staining of lung tissues. Examples of metastatic lesions are indicated by arrowheads. Insets show the enlarged area of the metastatic lesions. Magnification, 2.5 ×; Scale bar, 500 µm. Three independent experiments were performed in triplicate for assays shown in (D)–(G). Data are represented as the mean ± SEM; :*p* < 0.05; ::*p* < 0.01; :::*p* < 0.001; NS, not significant from Student's *t*‐test.

Recruitment of other cell types to prepare a favorable microenvironment for the survival and growth of disseminated metastatic cells at distant sites is a hallmark of the pre‐metastatic niche.^[^ ^25^ ^]^ Immunohistochemistry revealed positive *α*‐SMA staining in the metastatic lesions in the lungs of mice injected with MHCC97L CTL‐KD‐EVs but not in either untreated mice or mice injected with NID1‐KD‐EVs, suggesting that the activation of pulmonary fibroblasts induced by EVs is NID1‐dependent (Figure 6A). In accordance with the findings that S100A4‐positive fibroblasts induce an angiogenic microenvironment for metastatic colonization,^[^ ^26^ ^]^ NID1‐KD‐EVs upregulated the expression of S100A4 and promoted the growth of MRC‐5 human lung fibroblasts (Figure 6B and Figure S9A, Supporting Information). The uptake of EVs by MRC‐5 was detected after incubation with EVs (Figure 6C).

Cytokines, crucial regulators of cell–cell signaling, play critical roles in modulating the tumor microenvironment.^[^ ^27^ ^]^ To identify cytokines secreted by EV‐NID1‐stimulated lung fibroblasts, a cytokine array was employed to measure the expression of cytokines in MRC‐5 cells treated with CTL‐KD‐ or NID1‐KD‐EVs. Cytokines that are potentially involved in tumor microenvironment modulation were shortlisted (Table S2, Supporting Information). Upregulation of tumor necrosis factor receptor 1 (TNFR1) mRNA was detected in MRC‐5 cells stimulated with CTL‐KD‐EVs but not in cells treated with NID1‐KD‐EVs. A similar trend of soluble TNFR1 (sTNFR1) level in the conditioned medium of MRC‐5 cells was observed (Figure 6D).

Functionally, the conditioned medium of MRC‐5 cells was demonstrated to induce colony formation and promote migration and invasiveness of PLC/PRF/5 cells. The stimulatory effect was further enhanced when MRC‐5 cells were pretreated with CTL‐KD‐EVs. However, this effect was hindered when MRC‐5 cells were either pretreated with NID1‐KD‐EVs or incubated with anti‐TNFR1 antibody (Figure 6E–G). A consistent effect of MRC‐5 medium on Hep3B cells was observed (Figure S9B,C, Supporting Information). The crucial role of sTNFR1 in metastasis was further demonstrated by the largely attenuated MHCC97L‐EV‐ and MHCCLM3‐EV‐induced colonization of murine p53−/−; Myc hepatoblasts into the lungs of animals injected with anti‐TNFR1 antibody (Figure 6H–J and Figure S10, Supporting Information).

### 2.6 Levels of EV‐NID1 and Serum TNFR1 Correlate with Tumor Stage of HCC

**Figure 7:**
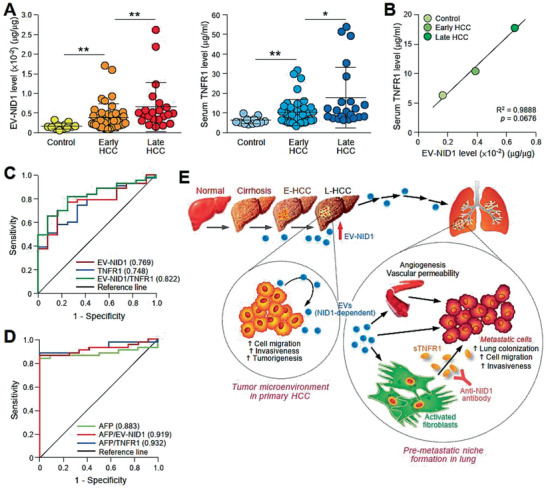
EV‐NID1 and serum TNFR1 levels correlate with the tumor stage of HCC. A) ELISA analysis of NID1 expression in circulating EVs obtained from sera collected from individuals without liver disease (Control) (*n* = 12), patients with early (*n* = 43) and late stage (*n* = 22) HCC (left). ELISA analysis of serum TNFR1 in the same subjects (right). ELISA was performed in duplicate. B) Correlation between EV‐NID1 and serum TNFR1 levels determined in (A) using Pearson correlation test. C) ROC curves of EV‐NID1, serum TNFR1, and combined EV‐NID1 and serum TNFR1 for discriminating control subjects and patients with early stage HCC. D) ROC curves of AFP, AFP in combination with EV‐NID1, or serum TNFR1 for discriminating control subjects and patients with early stage HCC. E) Proposed signaling mediated by EV‐NID1. The EV‐NID1 level increases with HCC development. EV‐NID1 derived from metastatic HCC cells promotes liver tumor development and distant metastasis to the lungs. EV‐NID1 increases pulmonary vessel leakiness, angiogenesis, and colonization of cancer cells to the lungs and activates pulmonary fibroblasts to secrete TNFR1, which in turn promotes HCC cell growth and motility. ROC, receiver operating characteristic. Data are represented as the mean ± SEM; :*p* < 0.05 and ::*p* < 0.01 from Student's *t*‐test.

In the mouse model with the implantation of MHCC97L cells, the levels of EV‐NID1 increased with the luciferase signal intensity, which reflects the tumor burden in the mice (Figure 3G and Figure S5A,B, Supporting Information). To further evaluate the potential application of EV‐NID1 as a biomarker for HCC detection, the circulating EVs obtained from non‐HCC control subjects and HCC patients with early and late stage disease were validated prior to the analysis of NID1 level (Figure S11, Supporting Information). As shown in Figure 7A, the results revealed EV‐NID1 levels in control subjects ranging from 0.0005 to 0.0032 µg µg^−1^ with a mean level of 0.0014 µg µg^−1^. Compared to control subjects, early stage patients showed a significantly higher overall level of EV‐NID1 (mean, 0.0038 µg µg^−1^; range, 0.0007–0.0172 µg µg^−1^; *p* = 0.0037). Late stage patients displayed an even higher EV‐NID1 level (mean, 0.0066 µg µg^−1^; range, 0.0013–0.0264 µg µg^−1^; *p* = 0.0072) than early stage patients. Serum TNFR1 showed concomitant upregulation with the HCC stages, and TNFR1 levels increased progressively from the control group (mean, 6.39 µg mL^−1^; range, 4.65–10.26 µg mL^−1^) to the early stage group (mean, 10.56 µg mL^−1^; range, 5.00–32.42 µg mL^−1^; *p* = 0.0079 vs control group) and late stage group (mean, 18.06 µg mL^−1^; range, 6.73–54.77 µg mL^−1^; *p* = 0.0179 vs early stage). The level of EV‐NID1 was well correlated with serum TNFR1 level (*p* = 0.0676) (Figure 7B). The positive association between HCC tumor stage and both EV‐NID1 and TNFR1 levels suggests the application of these molecules as noninvasive biomarkers for HCC.

Receiver operating characteristic (ROC) analysis was employed to evaluate the diagnostic value of EV‐NID1 and serum TNFR1 in HCC (Figure 7C). When comparing control subjects to early stage patients, analysis of EV‐NID1 resulted in an area under the curve (AUC) of 0.769 ± 0.068 with a 95% confidence interval of 0.636–0.903 (*p* = 0.0046). In addition, the AUC for serum TNFR1 was 0.748 ± 0.070 with a 95% confidence interval of 0.611–0.885 (*p* = 0.0091). These data indicated the effectiveness of EV‐NID1 and serum TNFR1 for discriminating HCC patients and control subjects. ROC analysis of combined EV‐NID1 and TNFR1 revealed greater sensitivity and specificity than either marker alone, with an AUC of 0.822. ROC analysis of alpha fetal protein (AFP), a biomarker of HCC, measured in the same cohort of sera revealed an AUC of 0.883. ROC analysis of combined AFP and EV‐NID1 or TNFR1 demonstrated an enhanced sensitivity and specificity than AFP alone (Figure 7D). These findings suggest that EV‐NID1 and TNFR1 together with AFP may be utilized as an effective biomarker for the early detection of HCC. Taken together, our results showed a progressive increase in EV‐NID1 levels during HCC progression. Mechanistically, EV‐NID1 promotes the formation of a pre‐metastatic niche by activating pulmonary fibroblasts to secrete TNFR1 to facilitate the colonization, growth, migration, and invasion of incoming HCC cells in the lungs (Figure 7E).

### 2.7 Antimetastatic Effect of TNFR1 Neutralizing Antibody as Potential Treatment for HCC

**Figure 8:**
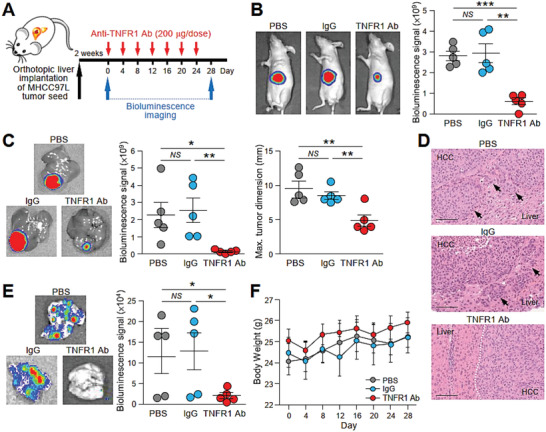
Treatment with TNFR1 neutralizing antibody effectively inhibits tumor growth and metastasis in mice implanted with metastatic tumor seed. A) Schematic diagram of the treatment regimen applied to mice implanted with luciferase‐labeled MHCC97L cells in the liver. Mice were administered PBS, IgG, or anti‐TNFR1 antibody (200 µg) via peritoneal injection every 4 days for 28 days (*n* = 5). B) Bioluminescence imaging of animals at the end of the experiment. Quantification of the luciferase signal is shown. The size of the liver tumors was measured and plotted. C) Ex vivo bioluminescence imaging of livers. Quantification of the luciferase signal is shown. D) Representative image of H&E staining of liver tissues showing the boundary of tumors obtained from (C). Dotted line indicates the bulging growth fronts of liver tumor. Arrows indicate the cluster of tumors nearby the liver‐tumor boundary. Magnification, 20 ×; Scale bar, 100 nm. E) Ex vivo bioluminescence imaging of lungs. Quantification of the luciferase signal is shown. F) Body weight of the mice was measured twice a week and plotted against time. Data are represented as the mean ± SEM; :*p* < 0.05, ::*p* < 0.01, :::*p* < 0.001 and NS, not significant from Student's *t*‐test.

Our findings showed that sTNFR1 was crucial to HCC metastasis; therefore, neutralization of serum TNFR1 could potentially block the communication between cancer cells and the target tissue microenvironment, leading to the suppression of metastasis. The therapeutic effect of the anti‐TNFR1 antibody was tested in mice implanted with metastatic MHCC97L cells in the liver (Figure 8A). Administration of anti‐TNFR1 antibody suppressed primary tumor growth compared to treatment with PBS or control IgG (Figure 8B,C). Histological examination revealed that liver tumors of the control and IgG group showed expansive tumor growth fronts, while bulging of the contour was observed in liver tumors of anti‐TNFR1 antibody‐administered mice (Figure 8D). Three out of five mice in both PBS and IgG group had metastasis to lungs in contrast to none of the mice treated with anti‐TNFR1 antibody showed distant metastasis to lungs (Figure 8E). It was noted that mice in all the experimental groups did not show signs of distress or significant changes in body weight (Figure 8F). Our findings provide preclinical evidence supporting the efficient blockage of oncogenic signaling mediated by HCC EVs using an anti‐TNRF1 antibody.

## 3 Discussion

EVs mediate intercellular communication via the transfer of their EV components to both neighboring and distant cells. The uptake of oncogenic EV contents leads to augmented aggressiveness of recipient cells. In HCC, tumor‐derived EVs have been shown to enhance the migratory ability and invasiveness of immortalized hepatocytes^[^ ^28^ ^]^ and induce cancer progression through promotion of epithelial‐mesenchymal‐transition (EMT).^[^ ^29^ ^]^ The transfer of HCC‐derived EV miRNAs to recipient cells promotes angiogenesis,^[^ ^30^ ^]^ cell motility,^[^ ^31^ ^]^ vascular permeability,^[^ ^32^ ^]^ and multidrug resistance^[^ ^33^ ^]^ and activates cell signaling.^[^ ^3^ ^]^ Indeed, our findings showed that HCC cells treated with tipifarnib, a farnesyltransferase inhibitor identified to be an inhibitor of EV biogenesis,^[^ ^18^ ^]^ displayed reduced EV secretion in vitro and formed smaller tumors in mouse liver, suggesting that EV release from HCC cells is crucial for tumor development and progression. The functionality of EVs is determined by the composition of their content. EV miR‐1247‐3p released by HCC cells has been shown to activate CAFs to foster lung metastasis.^[^ ^34^ ^]^ A recent study reported the effect of EV miR‐23a‐3p on HCC cells in attenuating antitumor immunity by upregulating PD‐L1 expression in macrophages.^[^ ^35^ ^]^ In addition to containing oncogenic miRNAs, EVs can also contain tumor suppressor miRNAs. For instance, miR‐451a, which inhibits HCC by inducing apoptosis and blocking angiogenesis,^[^ ^36^ ^]^ has been shown to be downregulated in circulating EVs from HCC patients miR‐451. Another tumor suppressor, miR‐1251/b, has been shown to inhibit tumor‐associated macrophage‐mediated cancer stemness in HCC cells.^[^ ^37^ ^]^ Tangible results have been reported on the functional diversity of EV miRNAs; nevertheless, the functional potential of EV proteins in HCC has not been well documented. Sugar metabolism‐regulated proteins have been described as differentially expressed proteins, with a fold change of 1.5, found in EVs of MHCC97L and MHCCLM3 cells when compared to in EVs of Hep3B cells.^[^ ^38^ ^]^ Although we used a fold change of 4 to distinguish differentially expressed proteins, most of the upregulated EV proteins that are involved in glycolysis, gluconeogenesis, and pentose phosphate pathway identified by the study are also found to be upregulated in our dataset. In addition, we also detected the presence of well‐known exosomal proteins such as Cav1, Met, and S100 family members in EVs of metastatic cells, as previously reported.^[^ ^28^ ^]^

Using proteomic profiling, we identified NID1 as a protein enriched in EVs from metastatic HCC cells. NID1, formerly known as entactin, is an essential structural component of the basement membrane and ECM. The strategic subcellular localization of NID1 enables it to mediate cell attachment and communication between cells and ECM. NID1 has been shown to activate the ERK/MAPK signaling pathway to promote EMT and chemoresistance.^[^ ^22^ ^]^ The functional capacity of NID1 in cancer metastasis has been revealed in breast, melanoma, ovarian, and endometrial cancers.^[^ ^22^, ^23^, ^24^ ^]^ In contrast, TMPRSS2‐induced invasion of prostate cancer cells has been shown to be mediated by NID1 degradation.^[^ ^39^ ^]^ NID1 secreted from endothelial cells inhibits breast cancer cell migration.^[^ ^40^ ^]^ Despite the evidence implicating the complex roles of NID1 in different cancers, its involvement in HCC remains unclear. Here, we showed for the first time the presence of NID1 in EVs of HCC cells, suggesting its unexplored functions in intercellular communications during hepatocarcinogenesis. Although NID1 has been found in EVs of melanoma cells, nasopharyngeal carcinoma cells, and urine of healthy individuals,^[^ ^41^, ^42^, ^43^ ^]^ yet their roles in human cancers and normal physiology have not been reported. In the current study, comprehensive functional characterization showed the capability of EV‐NID1 released by metastatic HCC cells to modulate the microenvironment in distant organs to support the growth and motility of disseminated HCC cells.

The enhanced expression of S100A4 in EV‐treated MRC‐5 cells and prominent *α*‐SMA staining in metastatic lesions in lungs of mice inoculated with HCC EVs indicate fibroblast activation by EVs derived from metastatic HCC cells. We further identified TNFR1 to be transcriptionally enhanced and secreted by MRC‐5 cells activated by EV‐NID1. At present, how NID1 activates the transcription of TNFR1 remains unknown. In silico analysis revealed the presence of two putative NF‐*κ*B binding sites in the promoter region −1517 and −1890 of TNFR1, suggesting the unexplored effect of NID1 in the activation of NF‐*κ*B pathway. The demonstration about the transcriptional regulation of TNFR1 by NF‐*κ*B induced by NID1 requires further investigation. TNFR1 signaling has been shown to perpetuate HCC tumor growth and tumor‐associated inflammation.^[^ ^44^ ^]^ Both full‐length and cleaved sTNFR1 are released by various cell types. Full‐length TNFR1, which is produced independent of cleavage by receptor sheddases, has been detected in EVs.^[^ ^45^ ^]^ The soluble form released by shedding of the ectodomain of TNFR1 has been shown to be mediated by ADAM17 proteolytic cleavage.^[^ ^46^ ^]^ Hypoxia and endoplasmic reticulum stress upregulate ADAM17 expression and therefore contribute to enhanced sTNFR1 release. However, the function of sTNFR1 has not been fully understood. ADAM17‐mediated shedding of TNFR1 in hepatocytes suppresses proapoptotic signaling during hepatic stress,^[^ ^47^ ^]^ and the increased level of sTNFR1 secreted by ADAM9‐null fibroblasts inhibits apoptosis of melanoma cells.^[^ ^48^ ^]^ In the clinical context, sTNFR1 levels are elevated in patients with glioblastoma and endometrial cancer.^[^ ^49^, ^50^ ^]^ In accordance with the mechanistic findings revealed in this study, the level of sTNFR1 progressively increases from non‐HCC individuals to patients with early stage HCC to patients with late stage HCC. In contrast, a higher level of sTNFR1 is associated with better survival in lung cancer patients with and without chronic obstructive pulmonary disease.^[^ ^51^ ^]^ In addition to its role in cancer, sTNFR1 is strongly associated with other diseases, such as cardiovascular mortality^[^ ^52^ ^]^ and nonrelapse mortality after hematopoietic cell transplantation.^[^ ^53^ ^]^

EVs are regarded as a molecular signature of the parental cells and provide insight about the origin and functions of the parental cells. Recent advances in liquid biopsies hold the promise of assessing EV content for clinical diagnostics. Thus, EVs are regarded as noninvasive sources of biomarkers for the detection and prognosis of various diseases. NID1 levels have been found to be higher in metastatic cancer patients than in patients without metastasis,^[^ ^24^ ^]^ and ovarian cancer patients have been shown to display an enhanced level of plasma NID1.^[^ ^54^ ^]^ A recent study reported the detection of NID1 in the saliva of patients with oral cavity squamous cell carcinoma (OSCC).^[^ ^55^ ^]^ The level of NID1 is well correlated with advanced stages of OSCC and poor survival of OSCC patients. Our study showed that the NID1 level in circulating EVs from mice increased progressively with the bioluminescence signal, which reflects the tumor burden in the animals. Furthermore, analysis of NID1 level in clinical samples revealed a progressive increase of EV‐NID1 level from control individuals to early and late HCC patients. With the recruitment of enough cases of patients with lung metastasis, the clinical relevance of EV‐NID1 in distant metastasis to lungs will be further investigated in the future. Together with our findings on serum TNFR1 levels in control subjects and HCC patients, ROC analysis revealed that EV‐NID1 and serum TNFR1 levels in combination may be a useful biomarker for HCC. It is intriguing to note that the NID1 transcript level showed no significant difference between nontumorous liver tissues from patient cohort and available from the TCGA database of liver cancer as well as other solid tumors such as colon, breast, and lung cancers (Figure S12, Supporting Information). According to the information of the Human Protein Atlas, the protein level of NID1 in liver, colon, and lung and breast cancers are undatable or weakly positive. It is worthwhile to understand how NID1 is particularly packaged and secreted in the form of EVs by cancer cells.

A preclinical study reported that administration of anti‐TNFR1 antibody in mice grafted with melanoma cells potentiates the effect of anti‐PD‐1 antibody in suppressing tumor growth. In the same study, TNFR1‐deficient mice injected with melanoma cells displayed a significantly enhanced anti‐PD‐1 response compared to that in wild‐type mice.^[^ ^56^ ^]^ Tumor necrosis factor (TNF) is an antitumor agent; however, the clinical application of TNF is limited by its induction of systemic cytotoxicity. The lethality of TNF in tumor‐bearing mice is mitigated by the application of anti‐TNFR1 antibody, thus facilitating the design of a safe TNF‐based antitumor therapeutic strategy.^[^ ^57^ ^]^ Here, we showed in a mouse model that anti‐TNFR1 antibody significantly suppressed liver tumor formation and distant metastasis to the lungs. HCC is often diagnosed at an advanced stage; therefore, most HCC patients are precluded from curative treatment options. Sorafenib is the first‐line therapy for patients with inoperable liver cancer; unfortunately, the ability of sorafenib to shrink tumors is modest, and its systemic toxicity is high. More importantly, most patients are highly refractory to sorafenib.^[^ ^58^ ^]^ New molecular targeted agents, such as regorafenib and lenvatinib, are not superior to sorafenib in terms of the overall survival of HCC patients. Supported by our current findings and previous studies about the crucial role of TNFR1 in tumorigenesis, blocking TNFR1 with neutralizing antibodies or antagonists either alone or in combination with other therapeutic agents may be envisaged to expand upon the current limited treatments for HCC.

## 4 Conclusion

In summary, our study demonstrated the functionality and clinical implications of EV‐NID1 in HCC. We also revealed that blocking EV‐mediated communication between cancer cells and the target tissue microenvironment with an anti‐TNFR1 antibody could diminish the malignant phenotype of HCC.

## 5 Experimental Section

##### Cell Culture

Human HCC cell lines, Hep3B and PLC/PRF/5 and other human cell lines, HUVEC line, MRC‐5 human lung fibroblasts and human 293FT, were purchased from American Type Culture Collection (ATCC) and cultured according to the ATCC recommendations. For other HCC cell lines, HLE was obtained from Japanese Collection of Research Bioresources (JRCB, Japan) and MHCC97L and MHCCLM3 were obtained from Cancer Institute, Fudan University, China. H2P and H2M were provided by Xin‐Yuan Guan, The University of Hong Kong, Hong Kong.^[^ ^59^ ^]^ Human immortalized normal liver cell lines were also used. LO2 was obtained from the Institute of Virology, Chinese Academy of Medical Sciences, Beijing, China and MIHA was provided by Jayanta Roy‐Chowdhury, Albert Einstein College of Medicine, New York.^[^ ^60^ ^]^ Murine p53−/−; Myc hepatoblasts was provided by Scott Lowe, Memorial Sloan Kettering Cancer Center, New York.^[^ ^61^ ^]^ These cell lines were cultured according to provider's recommendations. All cell lines were tested routinely before use to avoid mycoplasma contamination.

##### Isolation of EVs from Conditioned Medium of Cell Culture and Blood of Mouse and Patients

For EV isolation from cell culture supernatants, cells were cultured in media supplemented with 10% EV‐depleted fetal bovine serum (FBS). EV‐depleted FBS was prepared by overnight centrifugation at 100 000 × *g* at 4 °C (Beckman Coulter, Avanti JXN‐30). Supernatants were collected from cells cultured in medium with EV‐depleted FBS for 72 h and EVs were purified by differential centrifugation. In brief, culture supernatants were centrifuged at 2000 × *g* for 15 min to remove cell debris and dead cells (Thermo Fisher Scientific, Heraeus Multifuge X3FR). Microvesicles were removed after centrifugation at 20 000 × *g* for 30 min at 4 °C (Beckman Coulter, Avanti JXN‐30). Supernatants were first passed through 0.22 µm filter (Millipore) followed by centrifugation at 100 000 × *g* for 2 h at 4 °C (Beckman Coulter, Avanti JXN‐30) to pellet the EVs. The EVs were washed with PBS and collected by ultracentrifugation at 100 000 × *g* for 2 h at 4 °C (Beckman Coulter, Avanti JXN‐30). Mouse blood was obtained by cardiac puncture at the endpoint. Blood was also provided by non‐liver disease individuals and HCC patients. To collect circulating EVs, blood was first centrifuged at 1500 × *g* for 30 min to obtain serum (Thermo Scientific, Heraeus Multifuge X3R). Purification of circulating EVs from serum was performed using the ExoQuick PLUS Exosome Purification Kit for Serum & Plasma (System Biosciences). The serum was first centrifuged at 16 500 × *g* for 45 min (Eppendorf, 5430R) to pellet large vesicles. EVs were then purified using the purification kit according to manufacturer's protocol.

##### Validation of Isolated EVs

Proteins were extracted from isolated EVs and subjected to immunoblotting using anti‐Alix, anti‐CD9, anti‐TSG101, anti‐GM130, anti‐p62, and anti‐*α*‐tubulin antibodies. To examine the integrity of the isolated EVs, purified EVs suspended in PBS were dropped on formvar carbon‐coated nickel grids. After staining with 2% uranyl acetate, grids were air‐dried and visualized using Philips CM100 transmission electron microscope (FEI Company). The size range of EVs was measured by ZetaView BASIC NTA PMX‐120 (Particles Metrix GmbH).

##### Animal Studies

All animal studies were carried out under the research protocol CULATR 4394‐17, 4611‐18, 4847‐18 and 5012‐19 approved by the Committee of the Use of Live Animals in Teaching and Research (CULATR) at the University of Hong Kong. All animal work and procedures were followed strictly according to the Animals (Control of Experiments) Ordinance (Hong Kong) and the Institute's guidance from Centre for Comparative Medical Research, Li Ka Shing Faculty of Medicine, The University of Hong Kong. BALB/cAnN‐nu mice were used in experiments with animals. All mice were provided by and housed in specific pathogen free area in the Laboratory Animal Unit.

##### EV Education Model

Male 6 week old BALB/cAnN‐nu mice were injected intravenously with 15 µg EVs or PBS as control once per week for 3 weeks. At the end of education, mice were subjected to orthotopic liver implantation. To obtain tumor seed for orthotopic liver implantation, 1 × 10^6^ luciferase‐labeled MHCC97L cells were inoculated into the right flank of male 4 week old BALB/cAnN‐nu mice. After 2 weeks, mice were killed by euthanasia agent and tumor mass harvested was cut into small pieces of about 1 mm^3^ in size. Mice to be implanted with tumor seed were anesthetized and laparotomy was performed to expose the liver for tumor seed implantation. To monitor tumor development, mice which received intraperitoneal injection with D‐luciferin (GoldBio) were subjected to weekly bioluminescence imaging. Images were captured and the bioluminescence signal was quantified using IVIS Spectrum imaging system (Perkin Elmer). At the end of experiment, the mice were sacrificed and their lungs and livers were excised for histological analysis.

##### Labeling of EVs for Uptake Analysis

EVs were fluorescently labeled with either PKH67 or PKH26 Membrane Dye Labeling Kit (Sigma Aldrich) according to manufacturer's protocol. Labeled EVs were washed with PBS and collected by ultracentrifugation as described above. To examine uptake of EVs by cells, 1 × 10^5^ cells were treated with 10 µg labeled EVs for 24 h. After incubation, cells treated with EVs were fixed with 4% formaldehyde in PBS and stained with DAPI (4′,6‐diamidino‐2‐phenylindole) before examined under widefield fluorescence microscope (Leica) or laser scanning confocal microscopy (Carl Zeiss LSM700).

##### Tissue Distribution of EVs

Male 6 week old BALB/cAnN‐nu mice were injected intravenously with 15 µg CD63‐GFP^+^ EVs or PKH67‐labeled EVs. Each mouse was anesthetized and perfused to collect lung, liver, spleen, brain, and pancreas. Tissue sections from different organs were stained with DAPI and examined under confocal microscopy. Five random fields of each section were captured and three sections per organ were examined. Images were processed by ZEN software (Version 6.0.0.309) and the percentage of EV‐positive cells was quantified by ImageJ software (Version 1.50i).

##### Pulmonary Leakiness Assay

Male 6 week old BALB/cAnN‐nu mice were injected intravenously with 15 µg EVs or PBS as control. 20 h after EV injection, mice were injected intravenously with Texas Red lysine‐fixable dextran (70 000 MW, Thermo Fisher Scientific) at 100 mg kg^−1^. After 3 h, mice were injected intravenously with Alexa Fluor concanavalin A (Thermo Fisher Scientific) at 10 mg kg^−1^. 10 min later, each mouse was anesthetized and perfused with PBS and followed by 4% formaldehyde in PBS. Lung tissues were excised and immersed in 30% glucose in PBS overnight. Tissues were cryosectioned at 12 µm thickness. Tissue sections were stained with DAPI (Thermo Fisher Scientific) and examined under confocal microscopy for vascular leakage. Five random fields of each section were captured and three sections per lung were examined. Images were processed by ZEN software and the area of dextran was quantified by Image J software.

##### Lung Colonization Study

For lung colonization model, 1 × 10^5^ murine p53−/−; Myc hepatoblasts together with 10 µg EVs or PBS were injected intravenously into male 6 week old BALB/cAnN‐nu mice. The mice were subjected to weekly bioluminescence imaging. At the end of experiment, ex vivo bioluminescence imaging of lungs was performed, and dissected lungs were subjected to histological analysis.

##### Sample Preparation for Proteomic Analysis

Lysate in 8 m urea/100 × 10^−3^ m Tris‐HCl buffer was incubated at 60 °C for 10 min. Dithiothreitol (DTT) was then added to the samples at a final concentration of 5 × 10^−3^ m and incubated for 20 min at room temperature. Then iodoacetamide was added to a final concentration of 25 × 10^−3^ m and incubated in the dark for 30 min. Subsequently, trypsin was added at a ratio of 1:50 (trypsin:protein) after dilution of buffer to 1 m of urea and incubated at 37 °C for 16 h. The proteolysis was quenched by addition of 5% formic acid. The digested samples were desalted using C18 STAGE tips and concentrated by SpeedVac (Thermo Savant).

##### Liquid Chromatography Tandem Mass Spectrometry (LC‐MS/MS) Analysis

The protein digest samples were analyzed with an ultra performance liquid chromatography (UPLC)‐MS/MS setup. The analytical column was a 25 cm column (360 µm outer diameter, 50 µm inner diameter, 1.9 µm C18 packing material, Pepsep). The mobile phases were consisted of A (0.1% formic acid in water) and B (0.1% formic acid in 80% acetonitrile). Each sample (containing 2 µg peptides) (with technical triplicate) was loaded onto the analytical column by the auto‐sampler of the UPLC (EASY‐nLC 1200, Thermo Scientific) eluted with a gradient of 7% to 10% B for 20 min, followed by a gradient of 10% to 14% B for 30 min and subsequently eluted with a gradient of 14% to 27% B for 80 min then eluted with a gradient of 27% to 45% for 30 min at a flow rate of 200 nL min^−1^. For the MS analysis, Orbitrap Fusion Tribrid Mass Spectrometer (Thermo Scientific) was operated in a data‐dependent mode cycling through a high‐resolution (120 000 at 400 *m*/*z*) full scan MS^1^ (375–1500 *m*/*z*) followed by higher energy collision dissociation (HCD) MS^2^ scans on the most abundant ions from the immediately preceding full scan in a cycle time of 3 s. The selected ions were isolated with a 1.6 Da mass window and put into an exclusion list for 60 s after they were first selected for HCD.

##### Data Analysis

Raw files generated during LC‐MS/MS analysis were searched against the Uniprot Human database (Downloaded on 23 Mar 2020, 188 357 entries) with MaxQuant search engine (version 1.6.5.0). In which the search was specified to trypsin digestion (allowed up to two missed cleavages), oxidation of methionine as a dynamic modification, and iodoacetamide derivative of cysteine as a static modification. The mass tolerance for MS1 was 20 ppm for first search, 4.5 ppm for main search, and for MS2 was 20 ppm. With a decoy search strategy, the peptide false discovery rate (FDR) was set to 1%. Label‐free quantification (LFQ) option was enabled with normalization and only those proteins with nonzero LFQ intensities in all the three replicates were interpreted. Statistical analyses were performed with a two‐tailed Student's *t*‐test (MS Excel), with changes showing *p* < 0.05 considered as statistically significant. The mass spectrometry proteomics data had been deposited to the ProteomeXchange Consortium via the PRIDE^[^ ^62^ ^]^ partner repository with the dataset identifier PXD019566.

##### Enzyme‐Linked Immunosorbent Assay

Human NID1 ELISA Kit (Abnova) was used to determine NID1 expression in EVs extracted from sera of mouse and patients as well as cell culture medium. The isolated EVs were lyzed and the proteins were subjected to the measurement of NID1. Human AFP ELISA Kit (Solarbio) and Human TNFR1 (Sino Biological) were used to determine the level of AFP and TNFR1 in serum of patients. The level of EV‐NID1 was expressed as amount of NID1 over EV protein amount (µg µg^−1^) (w/w) and TNFR1 and AFP levels were expressed as TNFR1 and AFP amount per serum volume (ng mL^−1^).

##### Construction of NID1 Expression Plasmid

NID1 was expressed in the EVs of cells using XPack EV Protein Engineering Technology (System Biosciences). NID1 fragment (nucleotides 21–3123; Accession No. BC045606.1) was released from NID1/Entactin cDNA ORF Clone (Sino Biological) and subcloned into CMV‐XP‐MCS‐EF1*α*‐Puro Cloning Lentivector (System Biosciences) via XhoI and EcoRI sites. NID1 fragment (nucleotides 3124–3357) was amplified by polymerase chain reaction (PCR) using primers NID1‐3111F and NID1‐stopR using Human NID1/Entactin cDNA ORF Clone in cloning vector (Sino Biological) as template. Sequences of primers NID1‐3111F and NID1‐stopR were listed in Table S3, Supporting Information. The PCR fragment was purified, digested by restriction enzymes, and subcloned into CMV‐XP‐MCS‐EF1*α*‐Puro Cloning Lentivector carrying NID1 fragment (nucleotides 3124–3357) via EcoRI and PstI sites. The amplified NID1 region was confirmed by DNA sequencing.

##### Establishment of NID1 Knockdown and EV‐Targeting NID1 Stable Clones

MHCC97L NID1 knockdown stable clones (NID1‐KD1 and NID1‐KD2) were established using Human NID1 MISSION shRNA Plasmid DNA (Sigma‐Aldrich). Nontarget control clone (CTL‐KD) was generated using MISSIONTM nontarget shRNA control vector (Sigma‐Aldrich). FuGENE 6 Transfection Reagent (Promega) was used to transfect shRNA plasmid with the addition of MISSION Lentiviral Packaging Mix into HEK293FT cells. The viral supernatant was collected, centrifuged, and filtered. For the viral infection of MHCC97L, viral supernatant and polybrene transduction enhancer (8 µg mL^−1^) were added. 24 h after transduction, MHCC97L was selected by puromycin (Thermo Fisher Scientific). NID1 was expressed in the EVs of HLE and Hep3B by XPack EV Protein Engineering Technology (System Biosciences). XPack CMV‐XP‐MCS‐EF1*α*‐Puro Cloning Lentivector carrying Xpack‐NID1 fragment was used to establish EV‐targeting NID1 clone (XP‐NID1). Empty XP‐MCS‐EF1*α*‐Puro vector was used to establish vector control clone (XPack).

##### EV Treatment of MRC‐5 Cells

MRC‐5 cells were seeded at a density of 2 × 10^5^ in 6‐well plates and subjected to 72 h 10 µg EV treatment 1 day after seeding. After incubation with EVs, the cells were washed by PBS twice and cultured in complete medium for another 72 h. The conditioned medium was then used to treat HCC cells for different functional assays. HCC cells were treated with EVs for 72 h before subjected to functional assays.

##### Cytokine Array

Conditioned medium of MRC‐5 pretreated with PBS, MHCC97L CTL‐KD‐, and NID1‐KD‐EV were collected after 72 h in EV depleted medium. The collected conditioned media were incubated with Human Cytokine Array C1000 (RayBiotech) containing 120 human cytokine specific antibodies. Chemiluminescent signals were detected by ECL Western Blotting Detection Reagents. Relative cytokine intensities were normalized to the signal intensity of the control spots on the same membrane. Ratios among groups were calculated for the different cytokines by ImageJ software. Protein candidates were selected and confirmed by quantitative real‐time PCR analysis. Sequence of primers was listed in Table S3, Supporting Information and ELISA.

##### Treatment using Anti‐TNFR1 Antibody in Mouse Model

BALB/cAnN‐nu mice implanted with luciferase‐labeled MHCC97L tumor seed as described above was monitored using bioluminescence imaging. When the luciferase signal of mice reached 1.75 × 10^7^ ± 10%, they were randomized for treatment with PBS (200 µL), IgG antibody (200 µg in 200 µL PBS), or anti‐TNFR1 antibody (200 µg in 200 µL PBS) once every 4 days by intraperitoneal injection for a total of 28 days. At the end of the treatment, mice were subjected to bioluminescence imaging. The mice were then sacrificed and their lungs and livers were obtained for histological analysis.

##### Human Samples

Blood samples were collected from individuals with non‐liver diseases (as control subjects), and patients with early and late HCC who had not received any treatment. Information of blood donors was listed in Table S4, Supporting Information. The collection of blood samples was carried out at Queen Mary Hospital, Hong Kong and Zhujiang Hospital, Guangzhou, China. Informed consent was obtained from all donors. The collection and use of blood samples was approved by the Institutional Review Board of The University of Hong Kong/Hospital Authority Hong Kong West Cluster (HKU/HA HKW IRB) and Zhujiang Hospital of Southern Medical University. All experiments involving blood samples from all donors were performed in accordance with relevant ethical regulations.

##### Statistics

The data of all assays were calculated as mean ± standard error of mean (SEM). Student's *t*‐test performed by GraphPad Prism 6 was used for the statistical analysis. Pearson correlation test was used to analyze the correlation between EV‐NID1 and serum TNFR1 levels. The ROC curve was done to detect the AUC which reflected the accuracy of EV‐NID1 (µg µg^−1^) and TNFR1 (ng mL^−1^) alone or in combination with AFP (µg L^−1^) as diagnostic biomarkers to discriminating between the healthy control group from the early HCC group. The variables with *p* < 0.05 were analyzed by logistic regressions by IBM SPSS Statistics 25. *p*‐Value of less than 0.05 was considered as statistically significant.

## Conflict of Interest

The authors declare no conflict of interest.

## Author Contributions

X.W.M. and S.K.T. contributed equally to this work. Conceptualization: X.W.M., S.K.T., and J.W.P.Y.; Methodology: X.W.M., S.K.T., and J.W.P.Y.; Investigation: X.W.M., S.K.T., C.L.S.Y., E.M.L.K., Y.M.E.F., C.Y.S.C., H.P., M.P.W., C.O.N.L., T.K.W.L., V.M., and W.C.S.C.; Resources: L.Y.M., D.K.H.W., M.F.Y., J.C.M.H., P.C., X.X., and Y.G.; Writing—Original Draft: X.W.M., S.K.T., and J.W.P.Y.; Writing—Review & Editing: X.W.M., S.K.T., and J.W.P.Y.; Project Supervision: J.W.P.Y.; Funding Acquisition: X.W.M. and J.W.P.Y.

## Supporting information

Supporting Information

Supplemental Table 1
