# Supplementary material for: Nidogen 1‐Enriched Extracellular Vesicles Facilitate Extrahepatic Metastasis of Liver Cancer by Activating Pulmonary Fibroblasts to Secrete Tumor Necrosis Factor Receptor 1
Source: Adv Sci (Weinh). 2020 Aug 19;7(21):2002157. doi: 10.1002/advs.202002157 (PMC7640351; doi:10.1002/advs.202002157)
Supplement: Supplementary file 1 — Supporting Information [file ADVS-7-2002157-s001.pdf]

© 2020 Wiley-VCH GmbH

ADVANCED SCIENCE
Open Access

Supporting Information

for *Adv. Sci.,* DOI: 10.1002/advs.202002157

Nidogen 1-Enriched Extracellular Vesicles Facilitate Extrahepatic Metastasis of Liver Cancer by Activating Pulmonary Lung Fibroblasts to Secrete Tumor Necrosis Factor Receptor 1

*Xiaowen Mao, Sze Keong Tey, Cherlie Lot Sum Yeung, Ernest Man Lok Kwong, Yi Man Eva Fung, Clive Yik Sham Chung, Lung-Yi Mak, Danny Ka Ho Wong, Man-Fung Yuen, James Chung Man Ho, Herbert Pang, Maria Pik Wong, Carmen Oi-Ning Leung, Terence Kin Wah Lee, Victor Ma, William Chi-Shing Cho, Peihua Cao, Xiaoping Xu, Yi Gao, and Judy Wai Ping Yam**

Supporting Information

Nidogen 1-enriched extracellular vesicles facilitate extrahepatic metastasis of liver cancer by activating pulmonary lung fibroblasts to secrete tumor necrosis factor receptor 1

Xiaowen Mao, Sze Keong Tey, Cherlie Lot Sum Yeung, Ernest Man Lok Kwong, Yi Man Eva Fung, Clive Yik Sham Chung, Lung-Yi Mak, Danny Ka Ho Wong, Man-Fung Yuen, James Chung Man Ho, Herbert Pang, Maria Pik Wong, Carmen Oi-Ning Leung, Terence Kin Wah Lee, Victor Ma, William Chi-Shing Cho, Peihua Cao, Xiaoping Xu, Yi Gao, Judy Wai Ping Yam*

Supporting Experimental Section

Western Blot Analysis: Protein lysates were obtained by cell lysis with NETN lysis buffer (0.1% NP40, 25 mM Tris-HCl, 50 mM $NaCl_2$, 0.2 mM EDTA), supplemented with 10% cOmplete™ protease inhibitor cocktail and 10% PhosStop phosphatase inhibitor cocktail (Roche Applied Science). Bradford reagent (Bio-Rad Corporation) using bovine serum albumin as standard concentration was used to quantify protein amount. A total of 30 μg of protein per lane was resolved by SDS-PAGE, followed by a wet transfer to PVDF membranes (Amersham) for immunoblotting. Chemiluminescent signals were detected by ECL™ Western Blotting Detection Reagents (Amersham).

Immunohistochemistry and H&E: Formalin-fixed, paraffin-embedded tissue was sectioned with a thickness of 5 μm and deparaffinized in xylene, followed by rehydration in a gradient of alcohols (100%, 95% 70%) and distilled water. Antigen retrieval was conducted by immersing the sections in pre-heated EnVision™ FLEX Target Retrieval Solution, High pH (Agilent). The sections were microwaved for further 15 mins and allowed to cool down in

the retrieval solution for at least 20 minutes at room temperature. After blocking the endogenous peroxidase by EnVision™ FLEX Peroxidase-Blocking Reagent, the sections were subjected to incubation with primary and secondary antibodies. Signal detection was facilitated by the addition of Dako REAL EnVision™ Detection System and DAB chromogen for 30 mins and 2 mins respectively at room temperature. The specimen section was also stained with hematoxylin and eosin stain. NanoZoomer Digital Pathology System (Hamamatsu) was used to process slides and to create high-quality digital images for analysis.

Enzyme-Linked Immunosorbent Assay: Human CD63 ELISA Kit (Aviva Systems Biology) was used to determine CD63 expression in EVs extracted from sera of mouse and patients. The isolated EVs were lyzed and the proteins were subjected to the measurement of CD63. The level of EV-CD63 was expressed as amount of CD63 over EV protein amount (μg/μg) (w/w).

Tipifarnib Treatment of Cells: To examine the effect of Tipifarnib (AdooQ Bioscience) in EV secretion, MHCC97L was treated with 1 μM Tipifarnib for 48-hr. After replenished with EV-depleted medium, EVs were isolated from the conditioned medium of Tipifarnib-treated MHCC97L after 72 hr. The amount of isolated EVs was quantified. To study the ability of HCC cells with reduced EV secretion to form tumor, 1×10^6 Tipifarnib-treated MHCC97L cells were resuspended in 100% Matrigel and inoculated into left lobe of the livers of 6-week old male BALB/cAnN-nu mice. After 6 weeks post injection, mice were sacrificed and liver tumor volume was measured using a caliper and calculated using the formula: volume = $L \times W^2 \times 0.5$ (L, the largest diameter; W, the smallest diameter).

Treatment of Cells with EVs prior to functional assays: HCC and HUVEC cells were seeded at a density of 1×10^5 in 6-well plate and incubated with 10 μg EV for 72-hr after seeding. After incubation with EVs, the cells analyzed by functional assays.

Soft Agar Assay: Molten top agar (0.4% agarose, 2× DMEM, 20% FBS) mixed with 2×10^4 cells was overlaid on top of the bottom agar (1% agarose, 2× DMEM, 20% FBS) in 60-

mm culture plate. The cell cultures were allowed to grow for 3-4 weeks. At the end of the experiment, the number of colonies was counted under inverted light microscope. Five fields were randomly selected, and number of colonies was counted.

Migration and Invasion Assays: Transwell® Permeable Supports (Corning) were used in the migration and invasion assays. For invasion assay, BD Matrigel™ Basement Membrane Matrix (BD Bioscience) was used to coat the transwell inserts prior to seeding of cells. For both assays, 7×10^4 cells were resuspended in serum-free medium and seeded in the upper chamber of transwells. Medium with 10% FBS was added in the bottom chamber as chemoattractant. After incubation for a pre-determined period, cells that passed through the transwell membrane were fixed and stained with crystal violet. Four fields were randomly selected and number of migrated and invaded cells was counted.

Tube Formation Assay: Growth factor-reduced Matrigel (Corning) was allowed to polymerize in a 12-well plate and EV pretreated HUVEC cells were seeded at a density of 5×10^4 per well in Medium 200 (Gibco™). The number of capillary tubes formed was counted in 5 random fields under a 10× objective lens using Nikon Eclipse Ts2 microscope. Images of tube formation were analyzed by NIS-Elements L imaging software (Nikon Instruments Inc.).

In Vivo Angiogenesis: PLC/PRF/5 cells of 1.5×10^5 were mixed with 250 µl Growth factor-reduced Matrigel (BD Bioscience) at 4°C and injected subcutaneously (s.c.) into the back of BALB/cAnN-nu mice. Seven days after implantation, mice were sacrificed. Matrigel plugs were removed and subjected to H&E staining and immunohistochemistry staining of endothelial cell using anti-CD31 antibody (Abcam).

Quantitative Real-Time PCR Analysis: Total RNA was extracted from MRC-5 cells using RNeasy Mini Kit (QIAGEN) according to the manufacturer's instructions. Reverse transcription was performed using SuperScript™ VILO™ cDNA Synthesis Kit (Invitrogen). Real-time PCR was conducted using SYBR Green PCR Master Mix (Thermo Fisher Scientific) and performed on Applied Biosystems 7500 Fast Real-Time PCR System (Applied

Biosystems). The analysis was performed in triplicate and hypoxanthine guanine phosphoribosyl transferase I (HPRT) level was used as a reference gene for the normalization of cytokine expression in different samples. The expression of cytokine is expressed as copy number per HPRT. The fold change of cytokine expression in MRC-5 treated with CTL-KD-EV and NID1-KD1-EV relative to that in MRC-5 treated with PBS is also calculated.

Supporting Figures

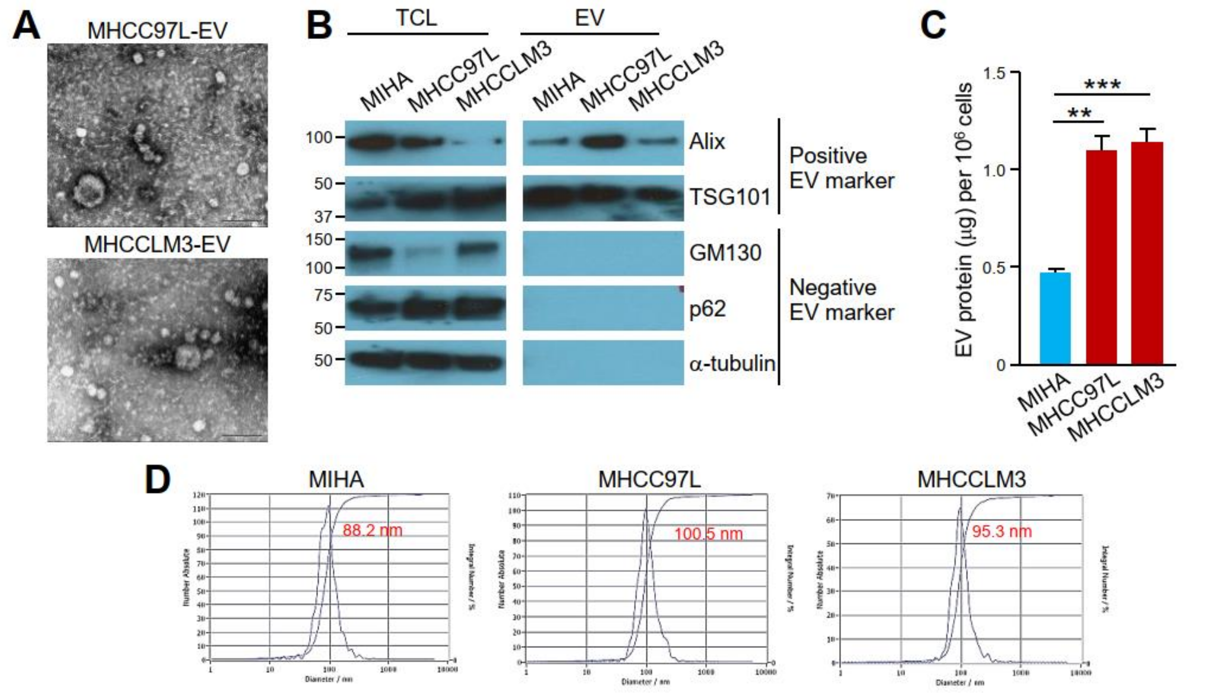

Figure S1. Characterization of EVs extracted from the conditioned medium of parental cell lines. (A) Representative electron micrograph of isolated MHCC97L and MHCCLM3 EVs evaluated by Philips CM100 Transmission Electron Microscope. Scale bar, 100 nm. (B) Western blotting of EV molecular markers in isolated EVs and total cell lysate (TCL) of cell lines. Positive EV markers include Alix and TSG101 while negative EV markers are *cis*-Golgi marker GM130, nucleoporin p62 and cytoskeletal α-tubulin. (C) Amount of protein extracted from EVs derived from the same cell number of different cell lines. The analysis was performed in triplicate and analyzed using Student's t-test. Data are represented as the mean ± SEM; ***P* < 0.01; ****P* < 0.001 (D) Size distribution of indicated EVs measured by ZetaView® BASIC NTA PMX-120 (Particle Metrix GmbH).

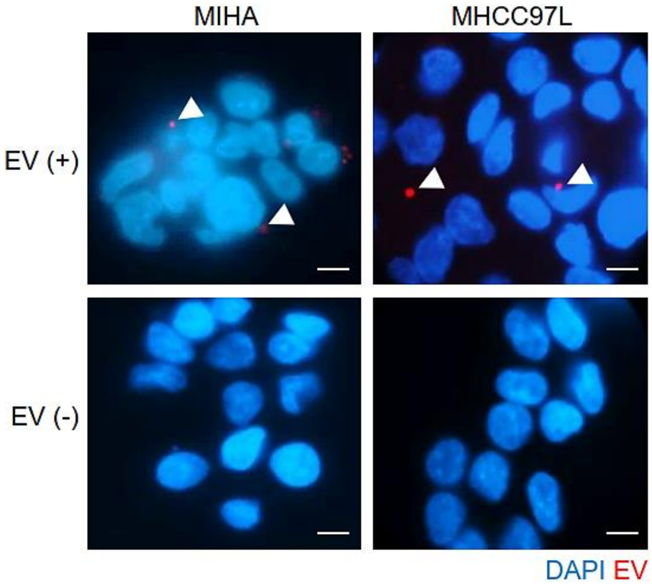

Figure S2. Uptake of EVs by cells in culture. Fluorescence microscopy of HCC cells incubated with or without PKH26-labeled EVs (red) for 24 hr. Representative images of fixed and DAPI-stained cells. The PKH26-labeled EV is indicated by arrowhead. Scale bar: 10 μm.

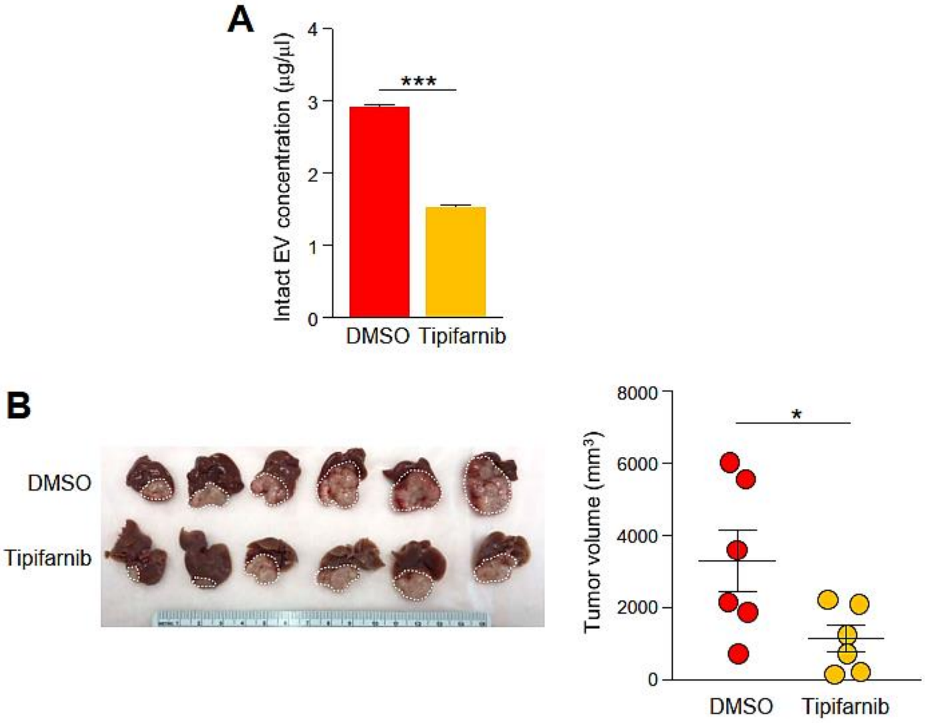

Figure S3. Reduction of EV secretion attenuates liver tumor formation. (A) Quantification of EVs isolated from conditioned medium of MHCC97L treated with 1 μM Tipifarnib or DMSO vehicle for 48 hr. The analysis was done in triplicate. (B) Image of dissected liver of mice subjected to orthotopic liver injection of Matrigel/MHCC97L (1×10^6 cells/injection) pretreated with 48-hr DMSO or Tipifarnib (1 μM) (n = 6). Measurement of tumor volume is shown. Data are represented as mean ± SEM. *$P < 0.05$ and ***$P < 0.001$ from Student's t test.

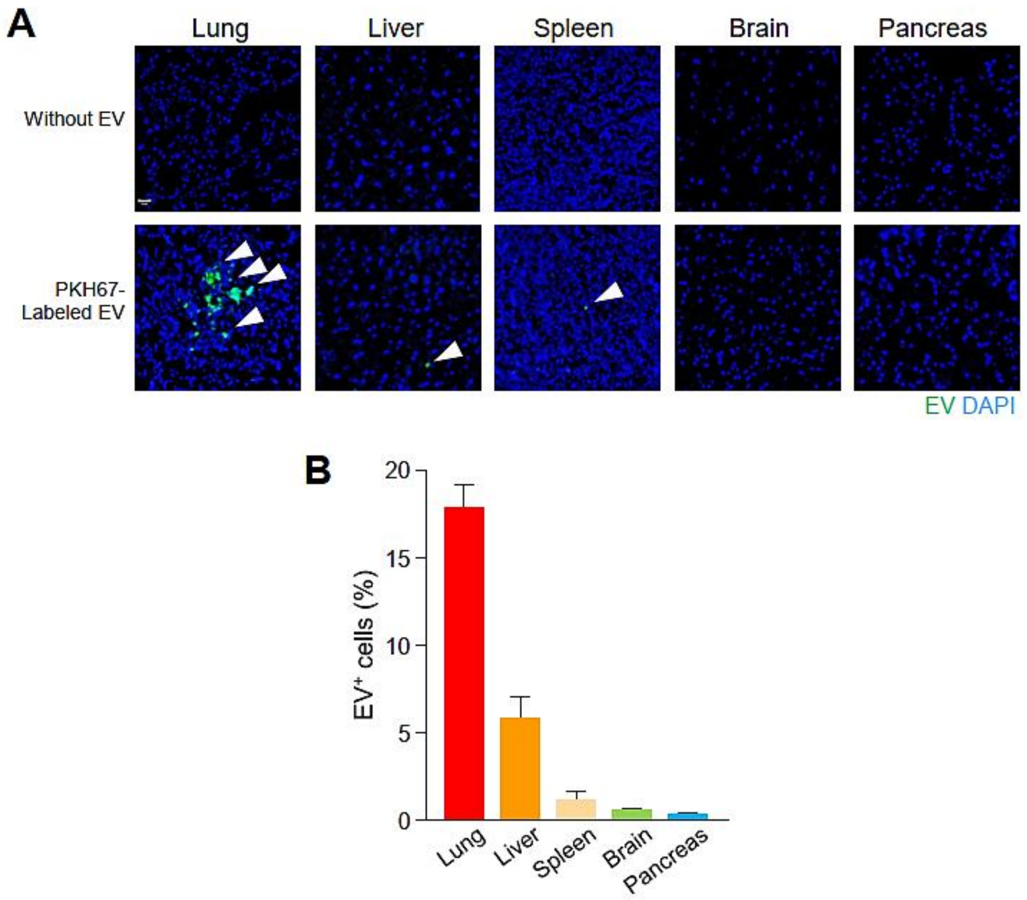

Figure S4. Tissue distribution of EVs after tail vein injection in mice. (A) Confocal microscopy of DAPI-stained tissue section dissected from mice subjected to euthanasia, perfusion and fixation 24 hr after intravenous injection with PKH67-labeled EVs (green). Green signal is indicated by arrowhead. Scale bar: 10 µm. (B) Quantification of signal in 5 random fields in each of 3 tissue sections per organ is shown. Data are represented as mean ± SEM.

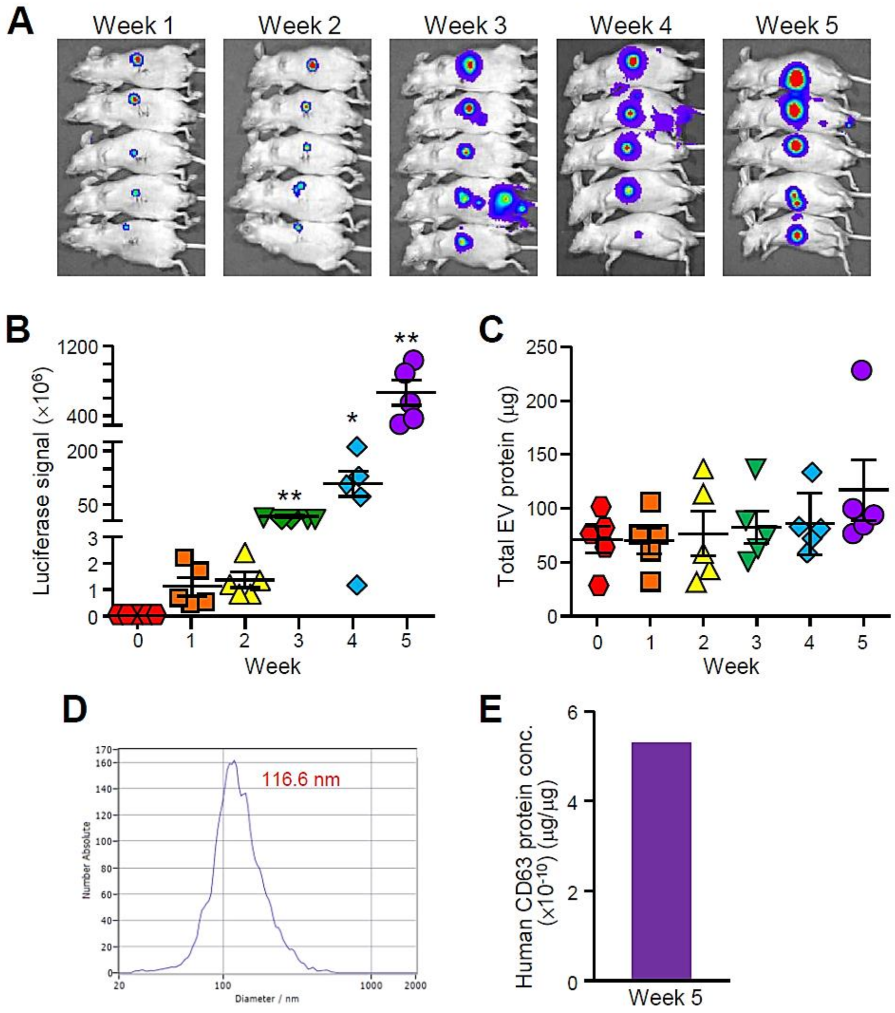

Figure S5. Quantification of circulating EVs obtained from mouse subjected to orthotopic liver implantation of HCC tumor seed. (A) Bioluminescence imaging of mice subjected to orthotopic liver implantation of tumor seed derived from luciferase labeled MHCC97L (n = 5). Images showing luciferase signal of animals from week 1 to 5. (B) Plot showing luciferase intensity increased with time. (C) Protein extracted from circulating EVs isolated from 250 µl serum weekly is shown. Quantification of protein was performed in triplicate. Data are represented as mean ± SEM. *$P < 0.05$, and **$P < 0.01$ from Student's t test. (D) Representative result showing the size distribution of circulating EVs isolated from blood of mouse at week 5 post implantation of tumor seed measured by ZetaView® BASIC NTA PMX-120 (Particle Metrix GmbH). (E) ELISA analysis of human CD63 expression in circulating EVs obtained from sera collected from mouse at week 5.

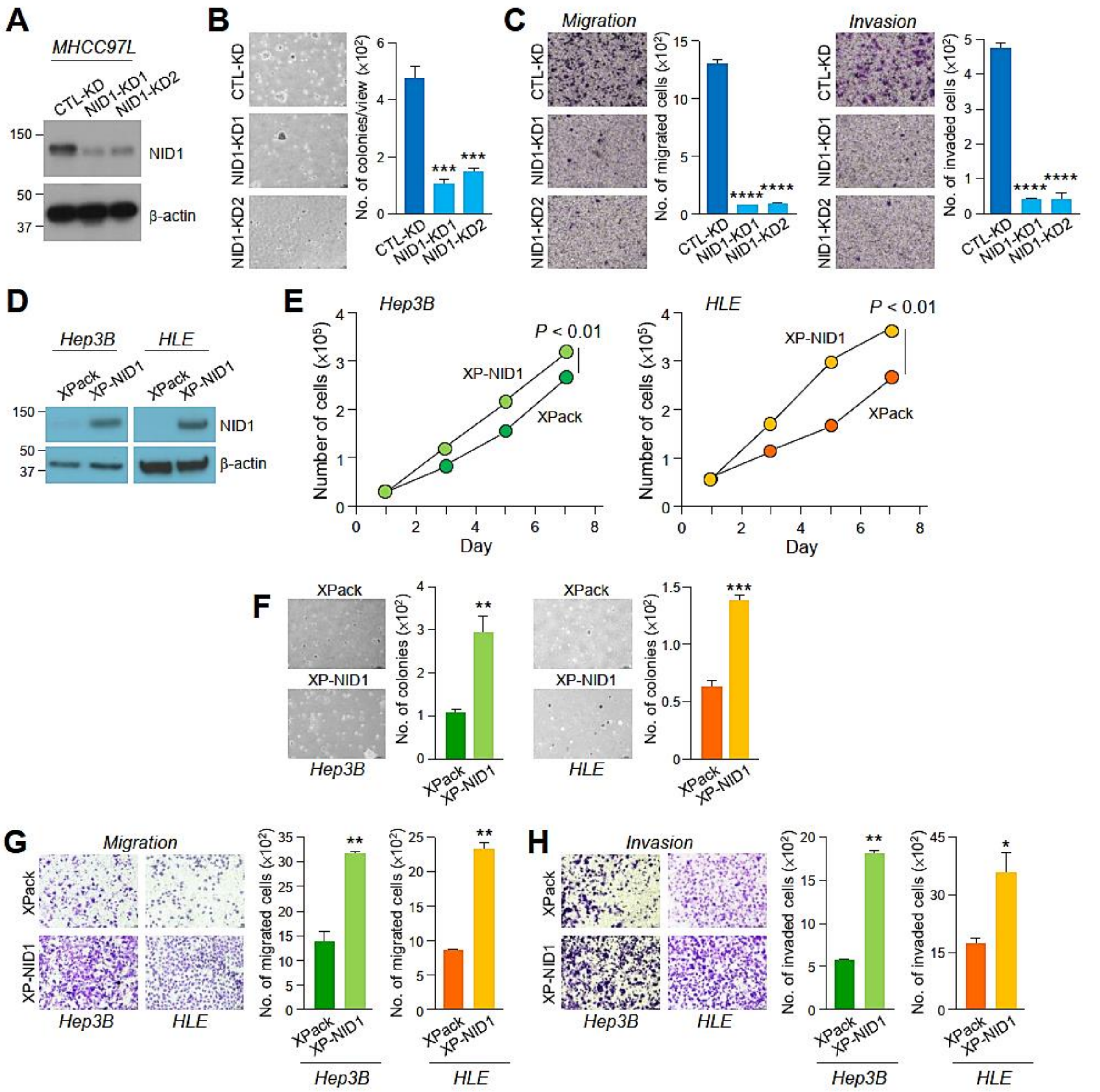

Figure S6. NID1 promotes HCC cell growth, migration and invasiveness. (A) Immunoblotting showing NID1 expression in NID1 knockdown clones (NID1-KD1 and NID1-KD2) and non-target control clone (CTL-KD) established in MHCC97L. (B-C) Migration and invasion assays using control and NID1 knockdown cells. (D) NID1 expression in stable control clone (XPack) and EV-targeting NID1 clone (XP-NID1) established in Hep3B and HLE analyzed by western blotting. (E) Proliferation curve comparing the growth rates of XPack and NID-XP cells. XPack and NID-XP cells subjected to soft agar assay (F) migration (G) and invasion (H) assays. Three independent experiments were performed in triplicate for all functional assays. Data are represented as mean ± SEM. *P < 0.05, **P < 0.01, ***P < 0.001 and ****P < 0.0001 from Student's t test.

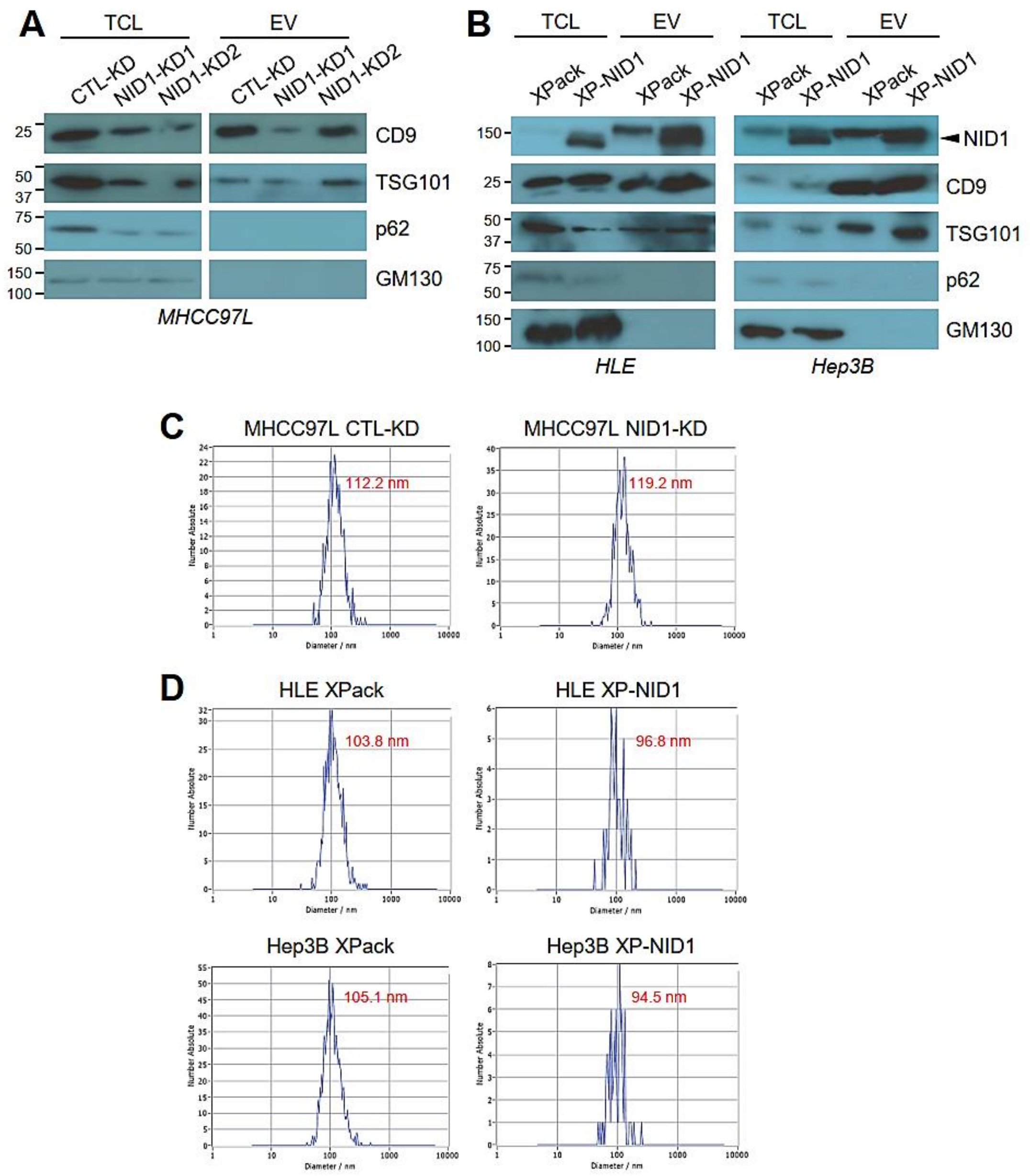

Figure S7. Characterization of EVs extracted from the conditioned medium of stable knockdown and overexpression clones of NID1. (A and B) Immunoblotting of EV markers of EVs isolated from the conditioned medium of MHCC97L control (CTL-KD) and NID1 knockdown (NID1-KD1 and NID1-KD2) cells, and control (XPack) and EV-targeting NID1 (XP-NID1) cells established in HLE and Hep3B. Positive EV markers include CD9 and TSG101 while negative EV markers are *cis*-Golgi marker GM130 and nucleoporin p62.Western blotting of EV molecular markers in isolated EVs and total cell lysate (TCL) of cell lines. (C and D) Size distribution of indicated EVs measured by ZetaView® BASIC NTA PMX-120 (Particle Metrix GmbH).

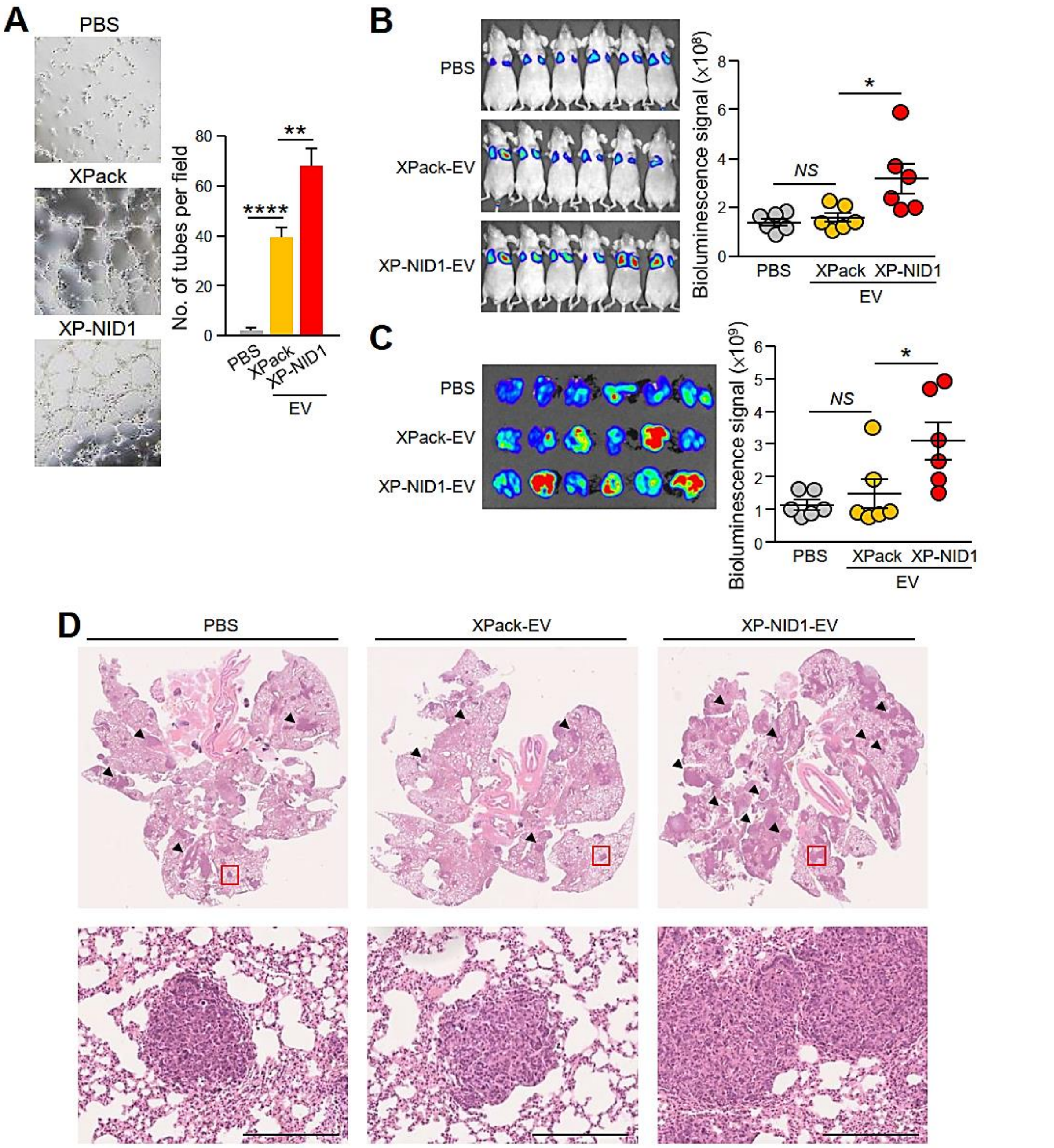

Figure S8. EVs engineered to express NID1 promote tube formation of endothelial cells and colonization of hepatoma cells in lungs. (A) Tube formation assay of HUVEC pretreated with EVs of Hep3B XPack or XP-NID1. Three independent experiments were performed in triplicate. Quantification of capillary-like tubular structures formed is shown. (B) Analysis of lung colonization of mouse p53-/-; Myc hepatoblasts (1×10^5) 2 weeks after co-injection with EVs of Hep3B XPack or XP-NID1 cells (10 µg) though tail vein (n = 6). Image of bioluminescence imaging of mice at the end of experiment. Quantification of luciferase signal is shown. (C) Bioluminescence imaging of dissected lung tissues. Quantification of luciferase signal is shown. (D) Representative images of H&E staining of lung tissues. Insets show the enlarged area of metastatic lesions. Magnification, 5×; Scale bar, 250 µm. Data are represented as mean ± SEM. **P* < 0.05, ***P* < 0.01, *****P* < 0.0001 and *NS*, not significant from Student’s t test.

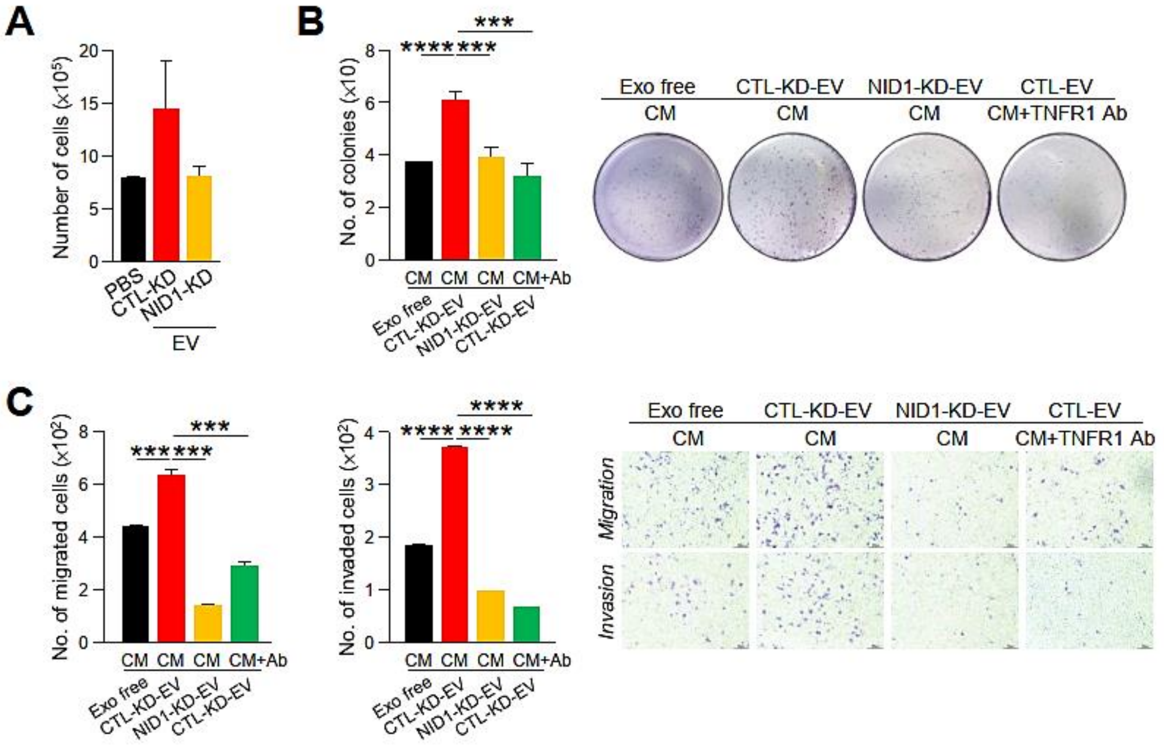

Figure S9. TNFR1 secreted by EV-NID1-activated pulmonary fibroblasts promotes colony formation, migration and invasiveness. (A) Quantification of the cell number of MRC-5 after treatment with MHCC97L CTL-KD- and NID-KD1-Exo for 72 hr. (B) Colony formation assay was performed using Hep3B incubated with the conditioned medium of MCR-5 pre-treated with 72 hours of incubation with EVs of MHCC97L CTL-KD or NID1-KD1 cells. Anti-TNFR1 neutralizing antibody is added to neutralize the activity of secretory TNFR1 (Ab) (0.4 µg/ml) in the conditioned medium. Quantification of the number of colonies is shown. Representative image showing fixed and crystal violet-stained colonies. (C) Migration and invasion assays performed using Hep3B incubated with the conditioned medium of MCR-5 pre-treated with the indicated EVs for 72 hours. Anti-TNFR1 neutralizing antibody (Ab) (0.4 µg/ml) is added to neutralize the activity of secretory TNFR1 in the conditioned medium. Quantification of the number of migrated and invaded cells is shown. Representative image showing fixed and crystal violet-stained migrated and invaded cells. Three independent experiments were performed in triplicate for all functional assays. Data are represented as mean ± SEM. ***P < 0.001 and ****P < 0.0001 from Student's t test.

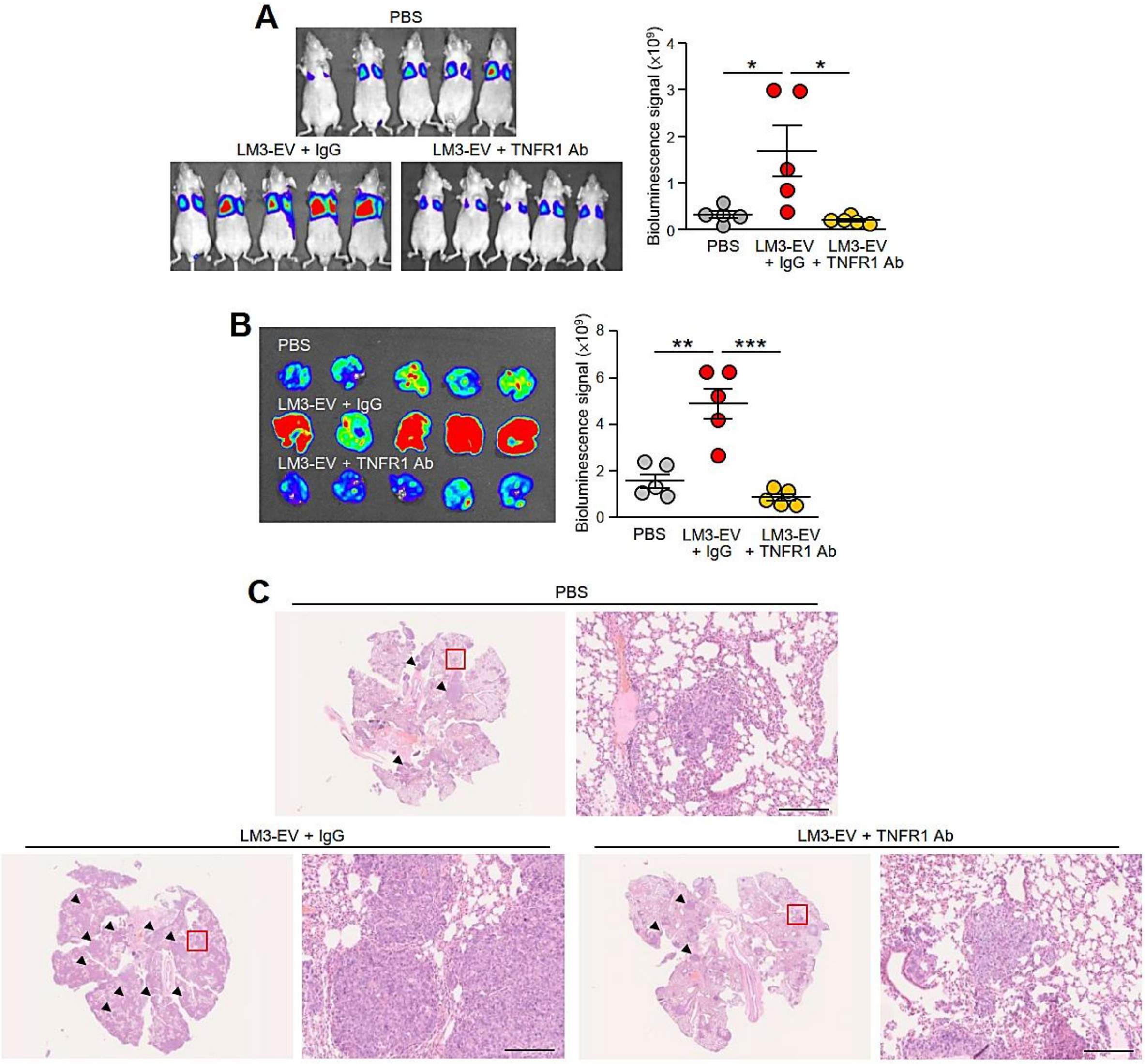

Figure S10. Anti-TNFR1 antibody neutralizes the promoting effect of MHCCLM3 EVs in the colonization of murine p53-/-; Myc hepatoblasts into the lungs of animals. (A) Bioluminescence imaging of mice (n = 5) subjected to intravenous coinjection of murine p53-/-; Myc hepatoblasts (1×10^5) with PBS, IgG (10 μg) or anti-TNFR1 antibody (TNFR1 Ab) (10 μg). Quantification of the luciferase signal is shown. (B) Ex vivo bioluminescence imaging of lung tissues. Quantification of the luciferase signal is shown. (C) Representative images of H&E staining of lung tissues. Examples of metastatic lesions are indicated by arrowheads. Insets show the enlarged area of the metastatic lesions. Magnification, 2.5×; Scale bar, 500 μm. Data are represented as the mean ± SEM; **P* < 0.05; ***P* < 0.01; ****P* < 0.001 from Student's t-test.

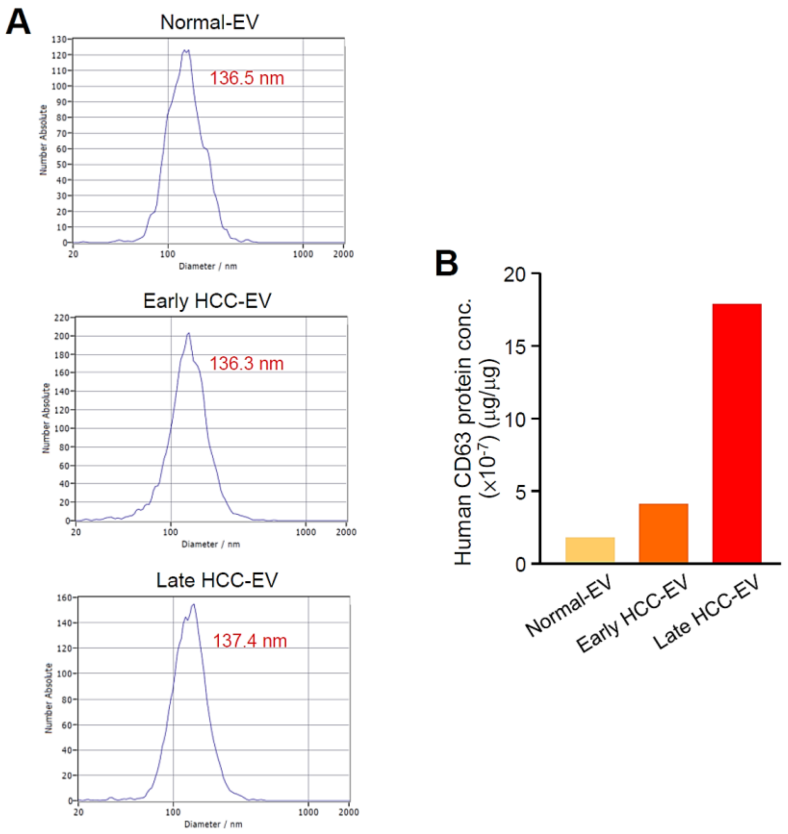

Figure S11. Validation of isolated circulating EVs. (A) Size distribution of circulating EVs isolated from control subjects and patients with early and late HCC measured by ZetaView® BASIC NTA PMX-120 (Particle Metrix GmbH). (B) ELISA analysis of CD63 expression in circulating EVs obtained from sera collected from individuals without liver disease (Normal-EV), patients with early (Early HCC-EV) and late stage HCC (Late HCC-EV).

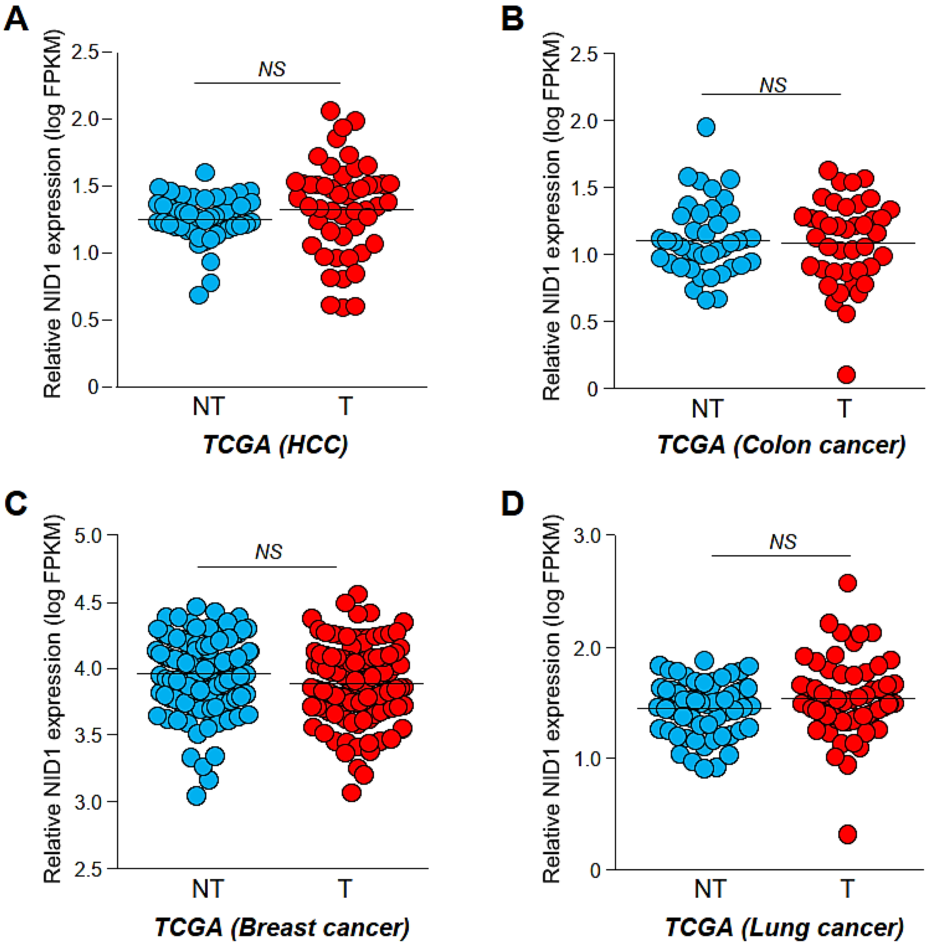

Figure S12. Expression of NID1 in human solid tumors. NID1 expression was evaluated using TCGA database of hepatocellular carcinoma (HCC) (A), colon (B), breast (C) and lung (D) cancers and analyzed by Student's t test. *NS*, Non-significant.

Supporting Tables

Table S1. Results of mass spectromatry produced by MaxQuant software.

(Attached as separate excel file)

Table S2. Analysis of cytokines secreted by MRC-5 cells pre-treated with EV-NID1 by quantitative real-time PCR.

	Copy number of cytokines per HPRT			Relative expression*	
**Cytokine**	**PBS**	**CTL-KD -EV**	**NID1-KD1 -EV**	**CTL-KD -EV**	**NID1-KD1 -EV**
IGFBP1	0.0045	0.0143	0.0323	3.16	7.11
SCFD1	1.0378	1.8918	2.0034	1.82	1.93
**TNFR1**	**3.7061**	**6.4708**	**3.9425**	**1.75**	**1.06**
TIMP2	16.6259	29.0202	15.4285	1.75	0.93
TNFSF11B	54.7792	80.5709	68.5219	1.47	1.25
IGF1	0.0086	0.0085	0.0054	0.99	0.63
IGFBP2	0.0409	0.0227	0.0639	0.56	1.56
TNFSF10D	1.9900	0.9493	0.9706	0.48	0.49
TGFB3	0.0316	0.0125	0.0321	0.40	1.02
LEP	0.0251	0.0098	0.0665	0.39	2.65
TNFSF14	0.0000	0.0000	0.0003	0.00	0.00
TNF-α	0.0000	0.0005	0.0077	0.00	0.00
ADIPOQ	0.0000	0.0001	0.0001	0.00	0.00

* The relative expression of cytokine in MRC-5 treated with CTL-KD-EV and NID1-KD1-EV is calculated in reference to that in MRC-5 treated with PBS.

Table S3. Oligonucleotides used in the study.

**Primer name**	**Oligonucleotide sequence 5' to 3'**
NID1-3111F	Forward: GATGAATTCCGTGGTTGCTC
NID1-stopR	Reverse: CTAGCTAGCTCATTTCTGTTCGATACAGTCAA
IGFBP1-F	Forward: TTGGGACGCCATCAGTACCTA
IGFBP1-R	Reverse: TTGGCTAAACTCTCTACGACTCT
SCFD1-F	Forward: ATGCAGCGTTAGCAGCTAGTG
SCFD1-R	Reverse: GGCCTGTTAATGGCACGATATG
TNFRSF1A-F	Forward: TCACCGCTTCAGAAAACCACC
TNFRSF1A-R	Reverse: GGTCCACTGTGCAAGAAGAGA
TIMP2-F	Forward: AAGCGGTCAGTGAGAAGGAAG
TIMP2-R	Reverse: GGGGCCGTGTAGATAAACTCTAT
TNFRSF11B-F	Forward: GCGCTCGTGTTTCTGGACA
TNFRSF11B-R	Reverse: AGTATAGACACTCGTCACTGGTG
IGF1-F	Forward: GCTCTTCAGTTCGTGTGTGGA
IGF1-R	Reverse: GCCTCCTTAGATCACAGCTCC
IGFBP2-F	Forward: GACAATGGCGATGACCACTCA
IGFBP2-R	Reverse: CAGCTCCTTCATACCCGACTT
TNFRSF10D-F	Forward: TACCACGACCAGAGACACC
TNFRSF10D-R	Reverse: CACCCTGTTCTACACGTCCG
TGFB3-F	Forward: ACTTGCACCACCTTGGACTTC
TGFB3-R	Reverse: GGTCATCACCGTTGGCTCA
LEP-F	Forward: TGCCTTCCAGAAACGTGATCC
LEP-R	Reverse: CTCTGTGGAGTAGCCTGAAGC
TNFSF14-F	Forward: ATACAAGAGCGAAGGTCTCACG
TNFSF14-R	Reverse: CTGAGTCTCCCATAACAGCGG
FGF6-F	Forward: TGGCTATTTGGTGGGGATCAA
FGF6-R	Reverse: GAAGAGGGCACTTCTCACTCC
TNF-α	Forward: CTTCTGCCTGCTG CACTTTGGA
TNF-α	Reverse: TCCCAAAGTAGACCTGCCCAGA
ADIPOQ-F	Forward: TGCTGGGAGCTGTTCTACTG
ADIPOQ-R	Reverse: TACTCCGGTTTCACCGATGTC
HPRT-F	Forward: CCTGGCGTCGTGATTAGTGAT
HPRT-R	Reverse: AGACGTTCAGTCCTGTCCATAA

Table S4. Information of blood donors.

Clinical parameters	Category	Number of donors
HCC patients		
Gender	Male	52
	Female	13
Age	< 50	21
	50 - 60	20
	> 60	24
pTMN staging	I and II	43
	III and IV	22
Control individuals		
Gender	Male	11
	Female	1
Age	< 50	3
	50 - 60	2
	> 60	7

* The TMN staging is based on the size of the tumor (T), the spread to nearby lymph nodes (N) and the metastasis to distant sites (M).
